# LC-MS based plant metabolic profiles of thirteen grassland species grown in diverse neighbourhoods

**DOI:** 10.1038/s41597-021-00836-8

**Published:** 2021-02-09

**Authors:** Sue Marr, Jos A. Hageman, Ron Wehrens, Nicole M. van Dam, Helge Bruelheide, Steffen Neumann

**Affiliations:** 1 Bioinformatics & Scientific Data, Leibniz Institute of Plant Biochemistry, Weinberg 3, 06120 Halle, Germany; 2 Institute of Biology/Geobotany and Botanical Garden, Martin Luther University Halle-Wittenberg, Am Kirchtor 1, 06108 Halle, Germany; 3 German Centre for Integrative Biodiversity Research (iDiv) Halle-Jena-Leipzig, Puschstr. 4, 04103 Leipzig, Germany; 4 Biometris, Wageningen University and Research, Droevendaalsesteeg 1, 6708 PB Wageningen, The Netherlands; 5 Molecular Interaction Ecology, Institute of Biodiversity, Friedrich-Schiller University Jena, Dornburger-Str. 159, 07743 Jena, Germany

**Keywords:** Secondary metabolism, Data processing

## Abstract

In plants, secondary metabolite profiles provide a unique opportunity to explore seasonal variation and responses to the environment. These include both abiotic and biotic factors. In field experiments, such stress factors occur in combination. This variation alters the plant metabolic profiles in yet uninvestigated ways. This data set contains trait and mass spectrometry data of thirteen grassland species collected at four time points in the growing season in 2017. We collected above-ground vegetative material of seven grass and six herb species that were grown in plant communities with different levels of diversity in the Jena Experiment. For each sample, we recorded visible traits and acquired shoot metabolic profiles on a UPLC-ESI-Qq-TOF-MS. We performed the raw data pre-processing in Galaxy-W4M and prepared the data for statistical analysis in R by applying missing data imputation, batch correction, and validity checks on the features. This comprehensive data set provides the opportunity to investigate environmental dynamics across diverse neighbourhoods that are reflected in the metabolomic profile.

## Abstract (summary)

**Table:** 

Measurement(s)	plant metabolite
Technology Type(s)	liquid chromatography-mass spectrometry
Factor Type(s)	Season • Diversity Level
Sample Characteristic - Organism	Anthoxanthum odoratum • Avenula pubescens • Centaurea jacea • Dactylis glomerata • Festuca rubra • Geranium pratense • Holcus lanatus • Knautia arvensis • Leucanthemum vulgare • Phleum pratense • Plantago lanceolata • Poa pratensis • Ranunculus acris
Sample Characteristic - Environment	grassland biome
Sample Characteristic - Location	Jena

Machine-accessible metadata file describing the reported data: 10.6084/m9.figshare.13288721

## Background & Summary

Plants respond and adapt to environmental changes in many ways. Some plant species, for example, possess physical defences to cope with herbivores and abiotic stress factors^1^. In addition, plants also produce chemicals as defence strategies. These plant metabolites provide a unique opportunity to explore these adaptations as the metabolic profile is known to reflect environmental changes^2–4^. Both the primary and the secondary metabolome are involved in the responses to biotic^5,6^ and abiotic factors^7–9^. However, especially secondary metabolites, which are not directly involved in the primary metabolism, play a key role in plant defence strategies^5,6,10–12^.

Furthermore, compared to primary metabolite profiles, secondary metabolite profiles are more species specific even in varying environments^13^. Previous studies showed that plants change the composition of their metabolic profile and alter the abundance and the number of specific compounds, such as phenolics and terpenoids^7,14^, while maintaining their distinctive profiles^13,15^. In field experiments, the impact of abiotic and biotic factors vary across the season^16,17^. These factors include, for instance, light, nutrients, water and herbivory^18^. Changes in these conditions may affect the plants’ metabolic fingerprint in yet uninvestigated ways. The investigation of these changes may provide insights into the mechanisms behind plant adaptation strategies.

Grasslands are an ideal study system to investigate the effects of plant community compositions on the plant metabolomic profiles. In these ecosystems, we find a relatively high number of fast-growing grass and herb species^19^. Species that share similar characteristics form functional groups (FG). Here, we distinguish between the two FG: grasses and herbs. Most studies focus on visible traits when investigating these two FG^20–22^. Visible traits, for example, are a useful tool to understand and predict ecological strategies and functions. They are also supporting the investigation of relationships between functional traits – that describe all measurable characteristics of a plant individual - and the individual plant performance^23–26^. However, the investigative power of combining metabolomics data with such trait data has already been demonstrated in other studies^14,15,27–29^.

**Fig. 1:**
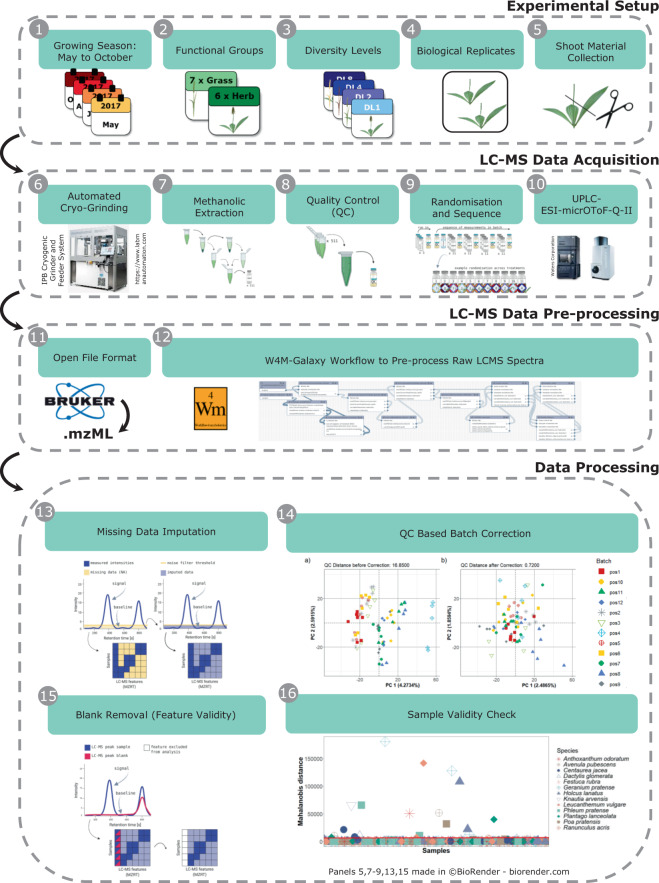
Data set overview. The data set includes the metadata of the experimental setup for the plant material collected in the TBE plots of the Jena Experiment (①-⑤), LC-MS raw data acquisition (⑥-⑩), data pre-processing steps (⑪-⑫), as well as data cleaning and validation (⑬-⑯). Created with BioRender.com.

**Fig. 2:**
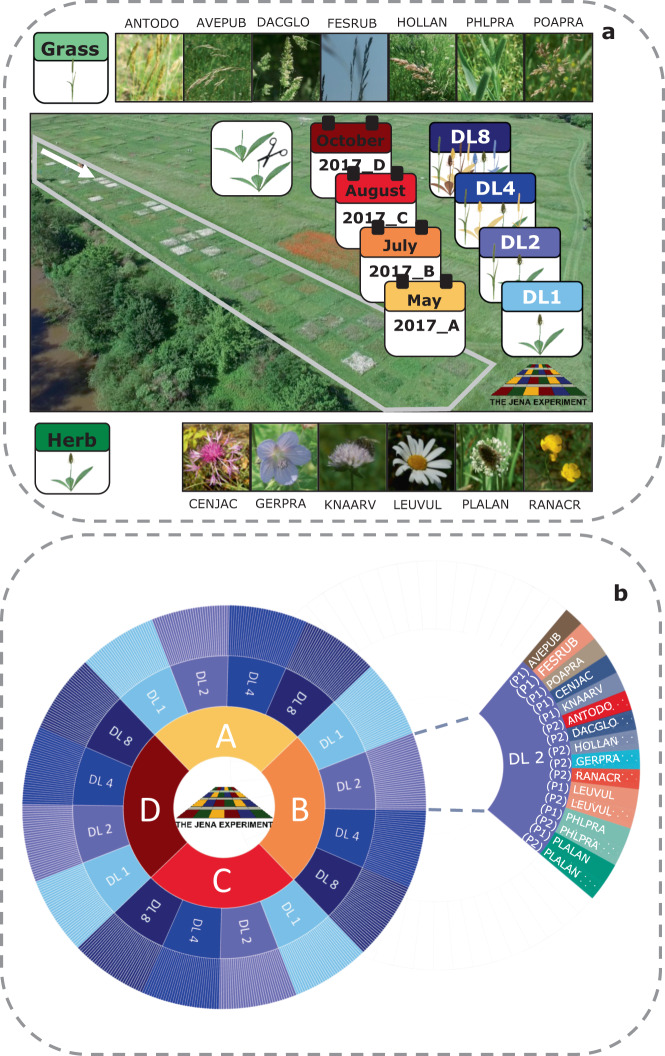
Experimental Design. (**a**) Plot and species overview. Plant material was collected in the plots of the TBE (grey borders) in the Jena Experiment. We collected shoots of seven grass (light green) and six herb species (dark green) in plots with four different diversity levels (DL). Here, either one (DL1), two (DL2), four (DL4) or eight (DL8) different species were grown per plot. In each plot, we harvested shoots of two replicates. The white arrow indicates the sampling direction, starting at the south end of the TBE. (**b**) Design overview. Plant material of species in both P1 and P2 were collected at four time points across the growing season in 2017 (May: A, July: B, August: C, October: D). The species pools P1 and P2 were each composed of four grass and four herb species. The three species LEUVUL, PHLPRA and PLALAN, were part of both pools. In total, we collected 512 *study samples*: 4 seasons x 4 DL x 2 Pool x 8 species x 2 replicates. For a detailed list of the species codes see Table 1.

In this data set, we collected plant material for metabolomic analysis in the field experiment “The Jena Experiment: Trait-Based-Experiment”, Germany^30^. An overview of the data set is provided in Table 1 and Fig. 1, including the experimental setup (①-⑤), metabolomic analysis (⑥-⑩), and the data processing (⑪-⑯). We recorded both visible traits and metabolomic profiles to investigate species specific responses of thirteen grassland species to the composition of their neighbourhoods. For the metabolomic analysis, we collected shoot material across the growing season in 2017 at four time points: May (A), July (B), August (C), October (D). We chose these time points to cover the whole growing season (May to October; Fig. 1 ①). The sown (target) species belonged to the FGs grasses and herbs (Fig. 1 ②, Fig. 2a). We investigated plants grown in communities with diversity levels (DL) composed of one (DL1), two (DL2), four (DL4) and eight (DL8) different species (Fig. 1 ③, Fig. 2). We collected shoots of two replicates per DL and species (Fig. 1 ④-⑤). For each species, we recorded characteristics of their surrounding neighbourhoods, including the number of plant species and their abundances per plot. For each sample, we recorded visible traits, such as the plant height, number of leaves and the level of damage caused by herbivory or pathogens.

**Table 1:**
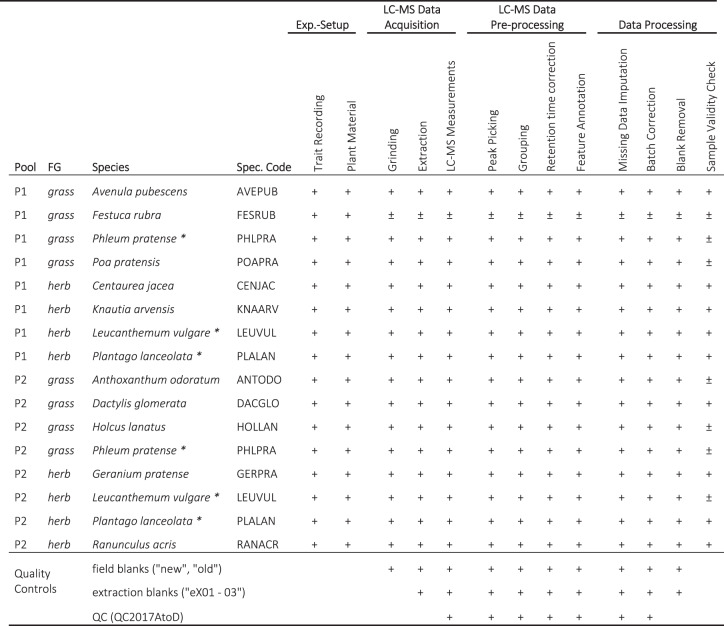
Steps of analysis performed on the thirteen target species and the quality controls.

Species belonging to the functional groups (FG) grass and herb were assembled in two groups of eight species (Pool). The Pools included four species per FG. Three of the species were represented in both pools (*). Shoots were collected at four time points (seasons: A, B, C, D) in four diversity levels (DL1, DL2, DL4, DL8). A detailed list of the study samples can be found in the associated Metadata Record (MTBLS679^33^). For details of the experimental setup, see Fig. 1, and Ebeling *et al*.^30^ for a plot overview. *Study samples* are processed in the respective analysis step (+). One sample was excluded from the analysis due to the loss of the sampled material, and some samples did not pass the final validation check (±; see section “Cryo Sample Preparation” and “Sample Validity Check”). This overview also indicates where the quality controls were used for the analysis.

In total, we collected 512 samples. For each sample, we acquired the metabolic profiles of methanolic extracts of the shoots on an Ultra Performance Liquid Chromatography coupled with an Electrospray Ionisation Quadrupole Time-of-Flight Mass Spectrometry (UPLC-ESI-Qq-TOF-MS; abbreviated to LC-MS in the following; Fig. 1 ⑥-⑩). We used quality controls (blanks and pooled extracts) to ensure data quality. We converted the acquired raw LC-MS data to an open file format (Fig. 1 ⑪) and processed them on the Galaxy-W4M infrastructure^31^. In Galaxy-W4M, we performed the feature detection, grouping and feature annotation (Fig. 1 ⑫). After this pre-processing, we prepared the data for statistical analysis. In R^32^, we performed missing data imputation, batch correction and validity checks on the LC-MS feature (Fig. 1 ⑬-⑯). In this data descriptor, we provide a detailed description of the analytical steps performed on the acquired LC-MS data and provide the comprehensive data set in the MetaboLights repository MTBLS679^33^.

## Methods

### Experimental setup

#### Experimental design

The Jena Experiment^34^ is a biodiversity ecosystem functioning experiment, designed to study plant and trait diversity effects on plant communities. The Jena Experiment is located in Jena, Germany, and includes the Trait-Based-Experiment^30^ (TBE; Fig. 2a). We collected plant material in the plots of the TBE. In the TBE, eight species selected from the functional groups (FG) grass and herb form a species pool. These Pools include four grass and four herb species^30^. Pool 1 (P1) comprises the grass species *Avenula pubescens* (AVEPUB), *Festuca rubra* (FESRUB), *Phleum pratense* (PHLPRA) and *Poa pratensis* (POAPRA) and the herbs *Centaurea jacea* (CENJAC), *Knautia arvensis* (KNAARV), *Leucanthemum vulgare* (LEUVUL) and *Plantago lanceolata* (PLALAN). Pool 2 comprises the grasses *Anthoxanthum odoratum* (ANTODO), *Dactylis glomerata* (DACGLO), *Holcus lanatus* (HOLLAN) and *Phleum pratense* (PHLPRA) and the herbs *Geranium pratense* (GERPRA), *Leucanthemum vulgare* (LEUVUL), *Plantago lanceolata* (PLALAN) and *Ranunculus acris* (RANACR). The target species of this study belonged to either P1 or P2 (Table 1, Fig. 2b). The three species *Leucanthemum vulgare*, *Phleum pratense*, and *Plantago lanceolata* were part of both pools.

In the TBE, the plant species are grown in plots with different diversity levels (DL): one (DL 1), two (DL 2), four (DL 4), and eight (DL 8) different species per plot (Fig. 2a). The plots are randomly distributed across the experimental site. P1 and P2 determine the plant species composition for each DL. Hence, all DL were composed of the species belonging to the respective Pool. For example, DL8 (P1) was composed of the following species: grass: AVEPUB, FESRUB, POAPRA, PHLPRA, herb: CENJAC, KNAARV, LEUVUL, PLALAN, while DL8 (P2) comprises these species: grass: ANTODO, DACGLO, HOLLAN, PHLPRA, herb: GERPRA, RANACR, LEUVUL, PLALAN. We collected the above-ground vegetative tissues of the thirteen target species. Per plot, we collected two plant individuals (replicates) at four time points in 2017. We chose dates across the growing season: May (A), July (B), August (C) and October (D). In total, we sampled 512 *study samples*: 4 seasons x 4 DL x 2 Pools x 8 species x 2 replicates (Fig. 1 ①-④, Fig. 2).

#### Traits & sampling

Prior to plant biomass collection in each season, we surveyed each plot to record the actual number of present species (species richness), both sown (target) and weed (not deliberately cultivated) species. We also estimated the abundance of each species (Shannon diversity) in relation to the plot size.

In each season, we collected the above-ground tissue of two replicates per plot and species (Fig. 2b). In each plot, we randomly chose two plant individuals as replicates from specimens with a similar phenological stage according to the BBCH^35^ scale. We recorded the following traits of these plant individuals: phenological stage (BBCH^35^), the number of leaves and inflorescences, plant height, and the proportional damage inflicted by either pathogen or mechanically.

The plants were cut 3 cm above the ground (Fig. 1 ⑤). An aliquot of shoot (leaf and stem) tissues was collected in plastic vials, snap-frozen on dry ice and stored for LC-MS analysis (referred to as *study sample*). The remaining biomass, including the inflorescences, was stored in plastic bags for biomass measurements. We collected the samples following the order of plots in the TBE (randomised DLs and Pools across the experimental site), starting at the southern end of the TBE^30^. We also recorded the exact time of the sampling for each sample to account for possible time-related shifts in the metabolic profile (sampling between 1 pm and 8 pm). We collected the samples within a single day to reduce the environmental influences to a minimum (for the exact dates see the MTBLS679^33^ data repository).

We applied the following labelling scheme to ensure the randomisation for sample extraction and LC-MS data acquisition. For each season, we assigned a number between 001 and 128 to each sample. These Lab-IDs were chosen randomly for each sample while collecting the biomass. For example, the Lab-ID *013_2017_A* refers to the sample 2017_A_*PHLPRA_A002_a*: collected in season 2017_A; *Phleum pratense*, in plot A002, which is referring to DL2 in P1, replicate a; and *013_2017_C* refers to the sample 2017_C_*FESRUB_B067_b*: collected in season 2017_C; *Festuca rubra;* in plot B067, which is referring to DL4 in P1; replicate b. The plot numbers (e.g. A002 and B067) and the corresponding DLs (e.g. DL2 and DL4) are specified in the sample metadata in the data records MTBLS679^33^. The sample preparation and extraction for the LC-MS data acquisition were conducted in the order of the respective Lab-IDs to ensure the equal distribution of seasons and full randomisation across the species, DL and replicates. Details on the randomisation can be found in the section “Sequence of LC-MS Measurements”. All details concerning the sampling strategy are included in the sample table in the MTBLS679^33^ data repository.

### LC-MS data dacquisition

#### Cryo sample preparation

We prepared the 511 *study samples* of frozen shoot material, collected in 20 mL vials, by adding two steel balls (7 mm) to the tubes. One sample tube (2017_B: FESRUB (P1): DL1_b) broke prior to analysis and was, therefore, excluded from further analysis. We used a cryo ball mill equipped with an autosampler (Labman IPB Cryogrinder Ball Mill, Labman Automation, Middlesbrough, UK) to grind the material at −75 °C for 150 s (5 cycles: 30 s grinding, 30 s pausing). We ground the samples according to their Lab-IDs and the season they were collected in (Fig. 1 ⑥).

#### Methanolic extraction

**Fig. 3:**
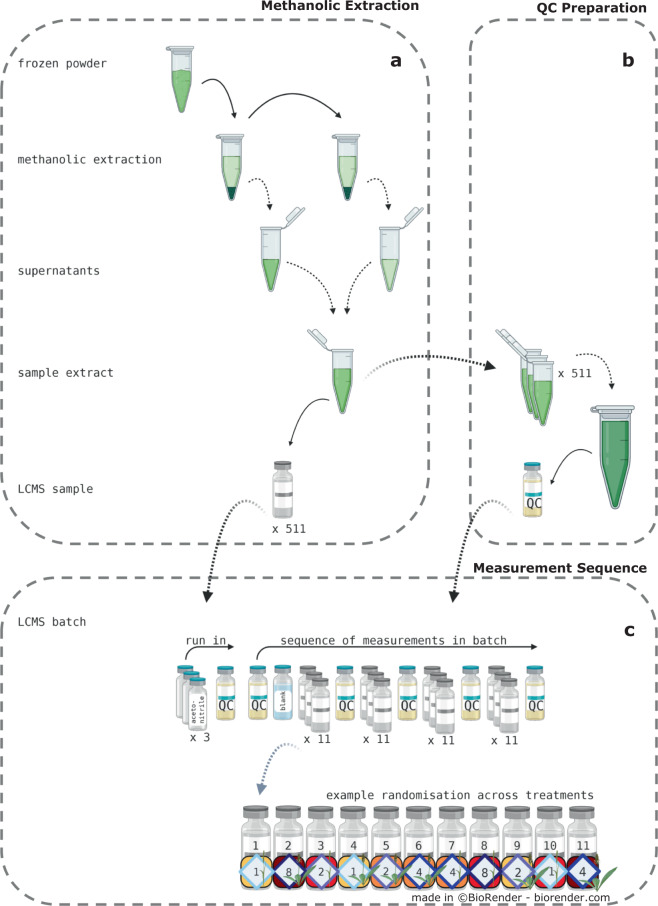
LC-MS sample extraction and sequence of measurements. (**a**) We prepared the 511 frozen study samples by grinding and extracting the resulting fine powder with methanol (note: one sample was lost prior to analysis, see “Cryo Sample Preparation”). For each sample, we combined the supernatants of two extraction steps to the sample extract. (**b**) We pooled aliquots of all 511 sample extracts and used them as the *Quality Control* (*QC*). (**c**) The LC-MS measurements were split into 12 *analytical batches*. Here, each batch measurement was led by a run-in sequence: 3 x acetonitrile, 1 x QC measurement. Per batch, samples were measured in four blocks, consisting of eleven *analytical samples*. The sample measurements were preceded by one *QC* measurement and one blank, and flanked by *QC* measurements. We randomised the 511 LC-MS samples (13 species, 4 seasons, 4 diversity levels) equally across the 12 batches. For treatment colour codes, see Fig. 2. Solid black arrows mark processing steps, while dashed black arrows indicate the transfer to another process. The dashed grey arrow indicates a zoom-in for clarification purposes. Created with BioRender.com.

We transferred aliquots (100 mg ± 50 mg) of the fine frozen powder to extraction tubes and added extraction beads (Rimax/Zircosil, 1.2–1.7 mm). For the extraction, we used methanol/water (80/20 v/v; HPLC-grade, Honeywell, Seelze, Germany) as the *extraction solvent*. We added the following internal standards at a 5 mM concentration to the *extraction solvent*: Kinetin (Roth, Karlsruhe, Germany), IAA-Val (Sigma-Aldrich, St. Louis, USA) and Biochanin A (Sigma-Aldrich, St. Louis, USA). The *extraction solvent* was added in a weight-specific five-fold surplus (Fig. 1 ⑦) to the frozen powder (e.g. 500 µL added to 100 mg powder), which we kept on liquid nitrogen. We thawed the prepared samples for 3 minutes at room temperature before extracting them in a homogeniser (Precellys® 24 Tissue Homogenizer, Bertin Technologies, Montigny-le-Bretonneux, France) for 90 s (2 cycles: 45 s run, 15 s pausing) at 6500 rpm. We centrifuged the extracts at 16168 g for 15 min and collected the supernatants in fresh extraction tubes (Fig. 3a). After an additional extraction of the remaining pellet, 160 µL of the combined supernatants were added to 40 µL of water/formic acid (99.9/0.1 v/v) (formic acid: VWR International, Radnor, USA) and stored at −20 °C for at least 48 hours (Fig. 3a).

To prepare the samples for mass spectrometry, we centrifuged the *sample extracts* at 16168 g for 15 minutes to remove particles. We transferred 160 µL of the resulting supernatant to vials equipped with 300 µL glass inserts (*analytical sample*). We extracted all *study samples* in batches of 44 samples, in the order of their Lab-IDs (e.g. one analytical batch contains *analytical samples* with the Lab-IDs 001 to 011 of season 2017_A, 2017_B, 2017_C, and 2017_D).

#### Quality controls

We used two types of blanks to account for possible contamination or inconsistency during extraction. The *field blanks* (plastic vials used for sampling) were included in the sampling, transportation and grinding steps. After the sampling in season 2017_A, we used a new shipment of plastic vials. We, therefore, labelled the *field blanks* “old” and “new” for the vials either used in 2017_A or 2017_B to 2017_D, respectively. We used the *extraction blanks* (eX01–03) to capture contaminations introduced in the methanolic extraction steps. For each replacement of *extraction solvent*, a new *extraction blank* was used. Both *field blanks* and *extraction blanks* were processed according to the extraction protocol applied to the *study samples*.

Furthermore, we pooled 10 µL aliquots of the 511 *sample extracts*, which we used as Quality Control (*QC*) throughout the LC-MS measurements (Fig. 1 ⑧; Fig. 3b).

#### Sequence of LC-MS measurements

We measured the 511 *analytical samples* in 12 analytical batches. Each batch was composed of an acetonitrile aliquot, a blank, a *QC* aliquot and 44 *analytical samples* (Fig. 1 ⑨, Fig. 3c). We distributed the *analytical samples* equally across the batches in the order of their Lab-IDs, and the season they were collected in (Lab-IDs were assigned to the samples randomly while sampling; see “Traits & Sampling”). For example, the *analytical samples* 2017_A_001 to 011, 2017_B_001 to 011, 2017_C_001 to 011, and 2017_D_001 to 011 were measured in batch “pos01”. We started the batch measurement sequence with three acetonitrile runs followed by the *QC*. After this run-in sequence, we measured the *QC* again, to equilibrate both the LC-column and MS-system, followed by one blank and a block of 11 *analytical samples* (Fig. 3c). We used the different blanks to detect potential systematic contaminations that were either introduced during sampling, extraction or the LC-MS measurements. After each block of *analytical samples*, we measured the *QC* again. The samples measured within one block were chosen randomly from the 44 samples assigned to the batch. After each batch, the MS ion source was cleaned, and the MS was recalibrated.

#### Analytical setup & data acquisition

We performed the data acquisition (Fig. 1 ⑩) on a liquid chromatography system (UPLC; ACQUITY UPLC System, Waters Corporation, Milford, USA) coupled with a mass spectrometer (ESI-Qq-TOF-MS; ESI-micrOTOF-Q-II, Bruker Daltonics, Bremen, Germany). Aliquots (2 µL) of the *analytical samples* were separated at 40 °C on an HSS T3 C_18_-column (1.8 µm, 1.0 × 100 mm, RP, Waters Corporation, Milford, USA) with the elution binary gradient at 0.15 mL min^−1^ flow rate: Solvent A (water/formic acid 99.9/0.1 v/v)/ Solvent B (acetonitrile/formic acid 99.9/0.1 v/v; acetonitrile: Merck, Darmstadt, Germany); initial: A 95%, 3 minutes linear A 82.7%, 10 minutes linear A 76%, 17 minutes linear A 5%, 18 minutes A 5%, 18.1 minutes linear A 95%, 20 min A 95%. We measured the ions in positive mode from 100–1000 m/z using the following instrument settings: capillary voltage 5000 V; nebuliser gas nitrogen; nebuliser 1.4 bar; dry gas nitrogen; dry gas temperature 190 °C; dry gas flow 6 L min^−1^; spectra rate 3 Hz; endplate offset: −500 V; Funnel 1 RF: 200 Vpp; Funnel 2 RF: 200 Vpp; in-source CID energy 0 eV; hexapole RF 100 Vpp; quadrupole ion energy 3 eV; collision gas nitrogen; collision energy 7 eV; collision RF 200/200 Vpp (timing 50/50); transfer time 58.3 µs; pre pulse storage 5 µs. We used an internal calibration (lithium formate clusters, 10 mM lithium hydroxide in isopropanol/water/formic acid, 49.9/49.9/0.2 v/v/v, at 18 min) for the normalisation of the measurements.

### LC-MS data pre-processing

We exported the vendor-specific data files (Bruker “.d”) using CompassXport (Bruker, version 3.0.9, http://www.bruker.com). The conversion of LC-MS raw data files to the open data format (“.mzML”)^36^ enables the data analysis in vendor-independent environments (Fig. 1 ⑪).

We pre-processed the raw LC-MS spectra of the *analytical samples* and the quality controls (blanks and *QC*) on the Galaxy-W4M infrastructure^31^ (based on XCMS 3.0). The workflow (10.15454/1.5640497789529167E12) includes the following analytical and processing steps: feature detection, grouping and retention time correction (Fig. 1 ⑫). A detailed description of parameter settings and tool versions used in the workflow is also shown in Table 2.

**Table 2:** Tools and Parameter used for pre-processing the LCMS raw data.

Tool name	Description	Version	Parameter	Value
MSnbase readMSData	Import mass-spectrometry data files	2.8.2.1		
findChromPeaks	feature detection	3.4.4.1	extraction method	40
			peak width (s)	5, 20
			signal to noise ratio	5
			prefilter	3, 100
			noise filter	100
xcms findChromPeaks Merger	merging xcms findChromPeaks	3.4.4.0		
xcms groupChromPeaks (group)	grouping of chromatographic peaks	3.4.4.0	method	PeakDensity
			bandwidth	6
			minimum fraction	0.75
			minimum number	1
			width of m/z slices	0.005
xcms adjustRtime (retcor)	retention time correction	3.4.4.1	method	PeakGroups
			minimum fraction	0.75
			maximum number	1
			smooth method	Loess – non-linear alignment
			degree smoothing	0.2
			family	gaussian
xcms groupChromPeaks (group)	grouping of chromatographic peaks	3.4.4.0	method	PeakDensity
			bandwidth	6
			minimum fraction	0.75
			minimum number	1
			width of m/z slices	0.005
CAMERA.annotate	Annotation of putative compounds	2.2.4	multiplier of sd	6
			general ppm error	5
			general abs error	0.005
			maximum ion charge	3
			maximum number	4
			isotope annotation	0.5
			correlation threshold	0.75
			grouping into pseudospectra	hcs
			correlation threshold	0.05
Check Format	Checking/formatting the sample and variable names	3.0.0		
Generic_Filter	Deleting samples and/or variables	2017.06	remove in “…” values upper	“rt”, 840 (s)
			remove in”…” values lower	“rt”, 80 (s)

The complete workflow is available in Galaxy-W4M (https://doi.workflow4metabolomics.org/W4M00008).

The initial step in the workflow is feature detection. The parameters were set in order to separate measured peaks from background noise (Table 2). We then grouped the features across samples and corrected them for retention time shifts. We grouped the corrected spectra again and annotated adducts and isotopes of the measured features.

After these pre-processing steps, we filtered the detected features for the region of interest (ROI). We cut features with retention times between 0 s to 80 s (injection peak and very polar compounds) and from 840 s to 1080 s (very nonpolar compounds). We exported the pre-processed data as separate data tables for sample metadata (*sampleMetadata*), variable metadata (*variableMetadata*) and the data matrix (*dataMatrix*), containing the measured intensities. These data matrices are also available in the associated metadata records MTBLS679^33^. The number of detected features per species is shown in Table 3.

**Table 3:** Number of unique LC-MS features (Fmeas) measured in both the *analytical samples* and the quality controls (Smeas).

FG	Species Code	Pre-processed		Validated	
		Smeas	Fmeas	Sval	Fval
	total	596	10252	499	10126
*grass*	ANTODO	32	1430	30	1310
*grass*	AVEPUB	32	1455	32	1358
*grass*	DACGLO	32	1281	32	1178
*grass*	FESRUB	31	1120	31	1020
*grass*	HOLLAN	32	1428	31	1317
*grass*	PHLPRA	64	1118	60	1054
*grass*	POAPRA	32	1046	29	924
*herb*	CENJAC	32	1711	32	1614
*herb*	GERPRA	32	1543	32	1464
*herb*	KNAARV	32	1708	32	1621
*herb*	LEUVUL	64	1384	62	1280
*herb*	PLALAN	64	1673	64	1581
*herb*	RANACR	32	1446	32	1348
*Quality Controls*	blank	12	126	0	0
	QC	73	5236	0	0

A feature is counted as part of the species when it is detected in at least 25% of the samples belonging to this particular species. After processing and blank removal, the remaining number of samples (Sval) and features (Fval) is used for analytical statistics.

## Data Records

A detailed description of the experimental setup, the performed analysis and the metadata of both *study samples* and the quality controls are available as MTBLS679^33^ “From Field to Feature in Ecometabolomics – LC-MS Based Metabolite Profiles of Thirteen Grassland Plant Species Reflecting Environmental Dynamics”. Raw data files of LC-MS analysis are also available in the repository. Furthermore, we provide data matrices of all stages of the processing steps (see Table 1).

The W4M-Galaxy history (10.15454/1.5640497789529167E12) that was used for data pre-processing is available at https://workflow4metabolomics.usegalaxy.fr/histories/list_published. All processing steps used for the data clean up are explained in the Supplementary File 1.

## Technical Validation

### Data processing

A detailed tutorial of the processing steps performed in R^32^ and the complete code used for data processing are provided as PDF and as R script in the MTBLS679^33^ repository. The tutorial PDF is also made available as supplemental material (Supplementary File 1).

#### Missing data imputation

In this study, the pre-processing of highly diverse LC-MS spectra lead to a data matrix with 90% zero values. This high number of zeros is a result of the data matrix containing all detected features, of which only small fractions belonged to a particular species (Table 3). Hence, features that are not part of the metabolic fingerprint in this species were not detected and are recognised as true zeros. Within a species, some features are only detected in a few specimens. These absences either occur due to variations in the technical performance or are indicators of actual biological adaptations to environmental changes. These are NA values, as the reason for their absence is uncertain at this stage of analysis. In the following, we refer to any missing values as *missing data*. In order to prepare the data matrix for further data cleaning and to make it accessible to processing and statistical analysis, we replaced the *missing data* with imputed values. Here, we imputed the *missing data* with random values (noise) by drawing absolute values from a normal distribution with mean 70 and a standard deviation of 20. We chose these values as they are below the threshold initially set for our data set, which equals 100 (Fig. 1 ⑬, see Table 2: feature detection). This choice is instrument specific and based on the prefilter parameters used in the pre-processing steps.

#### Batch correction

We performed a batch correction on the imputed data matrix. Splitting the 511 *analytical samples* into 12 analytical batches enhanced the chance of technical performance variability due to cleaning, recalibration and solvent replacements. These batch effects are mostly reflected in changes of intensities of the features across different batches. To account for these intensity shifts, the *QC*, which was measured multiple times across all batches (see “Sequence of LC-MS Measurements”), was used to determine the unwanted variation within (intra-batch distance) and between (inter-batch distance) batches. Ideally, the intensity profiles of the *QC* in all batches are identical. However, systematic variation between and within batches was present. Here, we used the *RUVs* function in the RUVSeq package (version 1.20.0)^37^, which is based on a principal component analysis (PCA), and applied it to the *QC* measurements (referred to as *pool* in *dataMatrix*). *RUVs* creates a PCA model of the systematic part of the variation of the *QC*. This PCA model describes unwanted systematic variation. In the next step, it substracts the PCA model from the study samples; thereby eliminating any unwanted systematic variation. A detailed description of the underlying calculations can be found in Risso *et al*.^37^.

The performance of the batch correction mainly depends on the number of components used for the analysis. We determined the optimal number of components to be used for the correction with a scree plot. In this scree plot, we compared the remaining inter-batch distances (Supplementary File 1 Fig. 1) after correction for different numbers of components. In this data set, the knee (or elbow) in the plot was reached after 6 components, as the inter-batch distances did not decrease anymore after 6 components (see Supplementary File 1 Table 3.2). After the batch correction, the calculated inter-batch distances for the *QC* measurements showed a strong decline (Table 4; Fig. 1 ⑭). The score plots before the batch correction show apparent batch effects in PC 1 and PC 2 (Fig. 1 ⑭). This shows that the batches, in which the *QC* has been measured, are the largest systematic source of variation for the *QC* measurements. After correction, the pattern in the PCs related to the different batches was no longer distinguishable. This shows that the huge variation of the feature intensities present in the original measurements related to the batches is removed and does not influence any consequent (statistical) analysis.

**Table 4:** Inter-batch distances calculated for both the *QC* (multiple measurements) and the *analytical samples* (single measurements).

Data	pre BC	post BC
*QC*	16.845	0.720
*analytical samples*	0.056	0.058

Distances are calculated before (pre BC) and after (post BC) applying the batch correction.

After performing the batch correction, the *QC* measurements are removed from both the metadata and data matrix (Table 1).

#### Blank removal

We checked the validity of the features before using them in the statistical analysis. We assigned a feature as valid when it was derived from an *analytical sample*. Here, we used the blanks as a reference for the validity check. Blanks did not contain a biological sample but were handled and processed like the *analytical samples*. Hence, we considered all features that were detected in blanks to be systematic contaminations introduced during sampling, extraction or the LC-MS analytical process. We removed all features that were detected in at least one blank from the data matrix and excluded them from any further analysis (Fig. 1 ⑮; see Table 3 for the number of features before and after the blank removal). Following this feature validity check, we also removed the blank samples from the sample metadata (Table 1).

#### Sample validity check

The amount of biological variation in the metabolomic profiles within a species differed across the species. This intra-species variation was found to be lower than the inter-species variation. To check the validity of each sample and, thereby, ensuring that the sample was not contaminated, we compared their metabolomic profiles to the average composition of their species. Here, we defined a feature as belonging to a species when it was detected in at least 8 of the samples (25%) in that species (Table 3). Note that for assigning a feature to the respective species, we used the data matrix without the imputed values (see “Missing Data Imputation”). As a quality measure, for each sample, we calculated Mahalanobis distances (Fig. 1 ⑯). We compared the distance of each sample to the average distance of the remaining samples in the respective species. For example, we calculated distances for the 32 samples in the species *Holcus lanatus* and compared the distance of the sample “HOLLAN (P2): 2017_A (DL4_b)” to the average distance of the other 31 samples. We kept only those samples that were closer than three times the average distance and shared over 25% of their features with their species (Table 1). Consequently, we excluded the following samples from further analysis as they did not pass the validity check: ANTODO (P2): 2017_B (DL8_b), 2017_C (DL1_b); HOLLAN (P2): 2017_A (DL4_b); LEUVUL (P2): 2017_D (DL4_a, DL4_b); PHLPRA (P1): 2017_D (DL1_b, DL8_b); PHLPRA (P2): 2017_D (DL8_a, DL8_b), POAPRA (P1): 2017_D (DL2_b, DL8_a, DL8_b).

#### Preparation for statistical analysis

After performing validity checks on the data, we prepared the cleaned and processed data matrix to be used for statistical analysis. The data matrix can be accessed in three different stages, with (1) imputed values or (2) zeros or (3) NAs for missing values (see “Missing Data Imputation”). Depending on the nature of the planned analysis, either one of the matrices can be used for statistical analysis and conclusion drawing.

## Usage Notes

This comprehensive data set provides the opportunity to investigate the metabolomic profiles on the feature level of thirteen grassland species grown in diverse neighbourhoods. The profiles were acquired from plants collected at different time point across the growing season. Therefore, relevant features and seasonality can be investigated within this eco-metabolomic dataset. Additionally, the mass spectrometry raw data are available in an open file format (mzML) and provide the opportunity to be re-processed with common metabolomics tools, such as xcms, OpenMS and MS-Dial.

## Supplementary information

Supplementary File 1

## Data Availability

The raw data files and processed data matrices are available in the online repository MTBLS679^33^. The complete history of the used workflow for the raw LC-MS data pre-processing is available in Galaxy-W4M^31^ from https://doi.workflow4metabolomics.org/W4M00008. We provide the complete R^32^ script used to process the data along with a detailed tutorial in the supplemental material (Supplementary File 1).
