# Supplementary material for: LC-MS based plant metabolic profiles of thirteen grassland species grown in diverse neighbourhoods
Source: Sci Data. 2021 Feb 9;8:52. doi: 10.1038/s41597-021-00836-8 (PMC7873126; doi:10.1038/s41597-021-00836-8)
Supplement: Supplementary file 1 [file 41597_2021_836_MOESM1_ESM.pdf]

From Field to Feature in Eco-metabolomics:

LC-MS based plant metabolic profiles of thirteen grassland species grown in diverse neighbourhoods

Marr, S., Hageman, J. A., Wehrens, R., van Dam, N. M., Bruelheide, H., Neumann, S.

December 12, 2020

Contents

1 Abstract **2**

2 Data Repository & Data Import **2**

2.1 Data Repository 2

2.2 Pre-processing in Galaxy-W4M 2

2.3 Data Import 3

3 Missing Data Imputation **5**

3.1 Missing Data Index 5

3.2 Filter Matrix 6

3.3 Imputation and Log Scaling 6

4 Batch Correction **8**

4.1 Preparation 8

4.2 Correction 10

4.3 Evaluation 11

5 Validity Check **17**

5.1 Feature Validity 17

5.2 Sample Validity 18

6 Processed Data Output **22**

6.1 Data Saving 22

6.2 Filter Matrix Re-calculation 22

7 Visualisation **22**

8 References **28**

List of Figures

1 W4M-Galaxy workflow for raw data pre-processing. 3
2 Number of principle components for batch correction 9
3 Inter-batch distances before and after batch correction 16
4 Sample validity check 20
5 Processed data visualisation 27

1 Abstract

In plants, secondary metabolite profiles provide a unique opportunity to explore seasonal variation and responses to the environment. These include both abiotic and biotic factors. In field experiments, such stress factors occur in combination. This variation alters the plant metabolic profiles in yet uninvestigated ways. This data set contains trait and mass spectrometry data of thirteen grassland species collected at four time points in the growing season in 2017. We collected above-ground vegetative material of seven grass and six herb species that were grown in plant communities with different levels of diversity in the Jena Experiment. For each sample, we recorded visible traits and acquired shoot metabolic profiles on a UPLC-ESI-Qq-TOF-MS. We performed the raw data pre-processing in Galaxy-W4M and prepared the data for statistical analysis in R by applying missing data imputation, batch correction, and validity checks on the features (detailed tutorial is included). This comprehensive data set provides the opportunity to investigate environmental dynamics across diverse neighbourhoods that are reflected in the metabolomic profile.

Our workflow includes the sample preparation, LC-MS measurements, the pre-processing of raw spectra and the data preparation for statistical analysis. The complete workflow is published in Marr et al. [1]

2 Data Repository & Data Import

2.1 Data Repository

The metabolomics data was acquired on a liquid chromatography system (ACQUITY UPLC System, Waters Corporation, Milford, USA; LC) coupled with a mass spectrometer (ESI-micrOToF-Q-II, Bruker Daltonics, Bremen, Germany; MS). In addition to the 512 analytical samples, this study includes quality controls, i.e. blanks and a *Quality Control* (*QC*) that was pooled of all samples. All raw data files are available in *MTBLS679 "From Field to Feature in Ecometabolomics: LC-MS Based Metabolite Profiles of Thirteen Grassland Plant Species Reflecting Environmental Dynamics" on https://www.ebi.ac.uk/metabolights/MTBLS679*. Detailed descriptions of the experimental setup, sample preparation, quality controls and raw data acquisition can be found in the related publication Marr et al. [1]

2.2 Pre-processing in Galaxy-W4M

Vendor-specific raw data files (.d) were converted to an open file format (.mzML) using CompassXport (version 3.0.9), enabling the usage across different data analysis procedures in vendor-independent environments. In this study, we used the Galaxy-W4M web service [2] (based on XCMS 3.0) to pre-process the raw data spectra as provided in *MTBLS679* in an automated workflow (Figure 1). The workflow enables both the processing of large data sets composed of diverse LC-MS spectra and the resulting diverse data matrix (*https://doi.workflow4metabolomics.org/W4M00008*). For all samples, including blanks and the *QC*, features were picked, grouped, corrected for retention time shifts and grouped again. Adducts and isotopes of the measured features were annotated using CAMERA. A detailed description of the workflow, including the parameter settings, is available in the corresponding manuscript by Marr et al.[1].

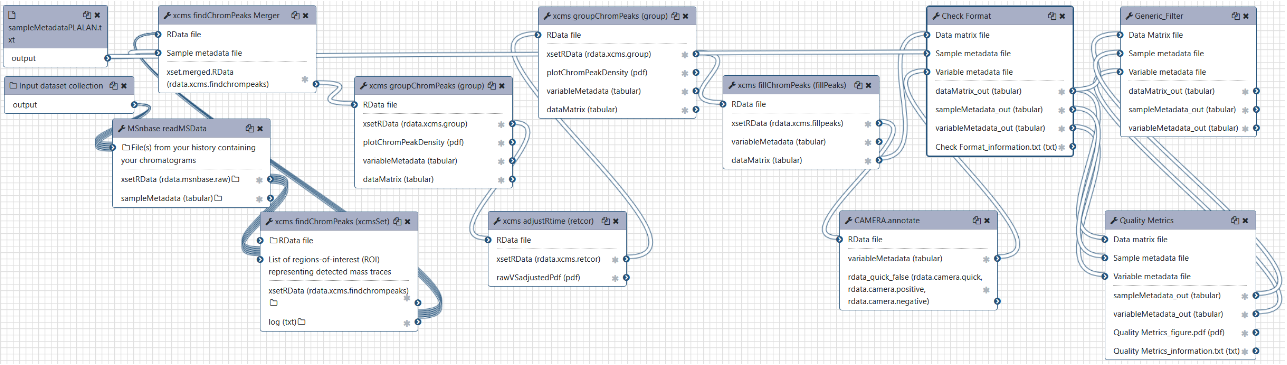

Figure 1: Galaxy-W4M workflow for raw LC-MS pre-processing. The pre-processing includes the following steps: peak picking, grouping, retention time correction, feature annotation, filtering for the region of interest. Parameter settings and tool versions are listed in Table 2 of the corresponding paper by Marr et al. [1]

2.3 Data Import

R Library

Load R libraries for data processing and visualisation. All libraries are referenced in section 8 References [5-14].

```r
# ---- Processing ----
library(BatchCorrMetabolomics)
library(magrittr)
library(RUVSeq)
library(StatTools)
library(vegan)

# ---- Visualisation ----
library(FactoMineR)
library(factoextra)
library(gridExtra)
library(ggsci)
library(ggtext)
```

Import

We import the data matrix (*dataMatrix*), including pre-processed LC-MS data of secondary metabolites in the shoots of grassland plant species, and the sample metadata (*sampleMetadata*), including environmental conditions, plant traits and LC-MS data acquisition, as provided in MTBLS679 *https://www.ebi.ac.uk/metabolights/MTBLS679* and *https://doi.workflow4metabolomics.org/W4M00008*. Note that we will refer to the data matrix and the sample metadata as *dataMatrix* and *sampleMetadata*, respectively, throughout the tutorial; regardless of their state of processing.

```r
# ---- Import Data from MTBLS679 ----
dataMatrix     <- data.frame(t(read.table(file   = "MTBLS679_dataMatrix.tsv",
                                            header = T, row.names = 1)))
sampleMetadata <- data.frame(read.table(file   = "MTBLS679_sampleMetadata.txt",
                                        header = T, row.names = 1, sep = "\t"))
```

```r
# ---- Matrix Example ----
dataMatrix[50:53, 405:408]
```

```
                                    M176T732 M176T296 M176T262 M177T237
pos_012_2017_A_GERPRA_B081_b_2.A.5_01_8474    0.000    0.000    0.000     0.00
pos_012_2017_B_CENJAC_B073_a_2.B.8_01_8466 2750.832    0.000    0.000 12730.26
pos_012_2017_C_KNAARV_B073_a_2.D.3_01_8494    0.000 4118.352    0.000     0.00
pos_012_2017_D_RANACR_B071_b_2.E.6_01_8455    0.000    0.000 1001.952 28523.63
```

```r
# ---- Metadata Example ----
sampleMetadata[50:53, c("injectionOrder", "batch", "Campaign", "desDiv")] #2, 4, 22
```

```
                                           injectionOrder batch Campaign desDiv
pos_012_2017_A_GERPRA_B081_b_2.A.5_01_8474           8474  pos2   2017_A      2
pos_012_2017_B_CENJAC_B073_a_2.B.8_01_8466           8466  pos2   2017_B      8
pos_012_2017_C_KNAARV_B073_a_2.D.3_01_8494           8494  pos2   2017_C      8
pos_012_2017_D_RANACR_B071_b_2.E.6_01_8455           8455  pos2   2017_D      2
```

For this example, we need only a few of the available factors in the sample metadata. Hence, we choose only relevant factors for processing and visualisation.

```r
# ---- Select Relevant Metadata: Factor ----
l1 <- c("class", "sampleType", "batch", "FuncGroup", "SpecCode", "desDiv", "Campaign",
    "Season")

# ---- Select Relevant Metadata: Numeric ----
l2 <- c("injectionOrder")
```

```r
# ---- Remove Remaining Variables From Metadata ----
delCol <- NULL
for (relVar in 1:ncol(sampleMetadata)) {
    lbl <- colnames(sampleMetadata)[relVar]

    if (lbl %in% l1) {
        idx1 <- which(colnames(sampleMetadata) == lbl)
        sampleMetadata[, idx1] <- as.factor(sampleMetadata[, idx1])
    } else {
        if (lbl %in% l2) {
            idx2 <- which(colnames(sampleMetadata) == lbl)
            sampleMetadata[, idx2] <- as.numeric(sampleMetadata[, idx2])
        } else {
            idx3 <- which(colnames(sampleMetadata) == lbl)
            delCol <- c(delCol, idx3)
        }
    }
}

sampleMetadata <- sampleMetadata[, -delCol]

# ---- Set Variable Types: Factor ----
w1 <- which(colnames(sampleMetadata) %in% intersect(l1, colnames(sampleMetadata)))

# ---- Set Variable Types: Numeric ----
w2 <- which(colnames(sampleMetadata) %in% intersect(l2, colnames(sampleMetadata)))
```

3 Missing Data Imputation

In this example, the simultaneous pre-processing of multiple species with diverse metabolomic profiles, cause the *dataMatrix* to incorporate a huge number of missing values. Those missing data can result from either variation in the instrument performances or actual biological reasons, such as species-specificity and adaptation to the environment. We prepare the *dataMatrix* for data cleaning and analysis by imputing all missing intensities, assuming that these features are independent of each other. We use random values picked from a normal distribution with a mean of 70 and an SD of 20. This range of values (70 ± 20) is instrument-specific and chosen in accordance with the prefilter for feature detection used in the pre-processing step (threshold were set to 100). For a detailed explanation also see section *Missing Data Imputation* in the corresponding manuscript by Marr et al. [1].

3.1 Missing Data Index

In order to preserve the missing intensities for the statistical analysis and investigation of the underlying biological question, we create an index for later reference, replace these zero values with NAs and create the *filterMatrix*.

```r
# ---- Save Reference ----
metaPre  <- sampleMetadata
Xall     <- dataMatrix

# ---- Define Index for Missing Data ----
NAidx    <- Xall == 0

# ---- Replace Zeros with NAs ----
XallZero <- Xall
XallNA   <- Xall
XallNA[Xall == 0] <- NA
```

3.2 Filter Matrix

We use the *filterMatrix* to subset the *dataMatrix* species-wise. Only those features are assumed to be present in a species that were detected in at least 25% of the samples belonging to this species. The values are calculated as the proportional occurrence of each feature in a particular species. Thereby, the number of samples with measured intensities for each feature is counted in relation to the total number of samples within this species (e.g. 0 = not present in this species, 0.25 = present in 25% of all samples in the species, 1 = present in all samples of the species). Note that in the *filterMatrix* the *QC* is referred to as *pool*.

```
# ---- filterMatrix Calculation ----
uniqLvl      <- unique(metaPre$SpecCode) %>% as.character() %>% sort()
nSpecies     <- length(uniqLvl)
filterMatrix <- matrix(ncol=ncol(XallNA), nrow=nSpecies)

colnames(filterMatrix) <- colnames(XallNA)
rownames(filterMatrix) <- uniqLvl

for (s in 1:nSpecies) {
  sp                <- uniqLvl[s]
  Xsp               <- XallNA[metaPre$SpecCode == sp,]
  metaSp            <- metaPre[metaPre$SpecCode == sp,]
  filterMatrix[s,] <- apply(Xsp, 2, function(x) sum(!is.na(x)))/nrow(Xsp)
}

# ---- filterMatrix Example ----
round(filterMatrix[ ,1:5], digits = 4)
```

```
        M127T743 M131T466 M132T149 M133T212 M133T467
ANTODO    0.0312   0.0000   0.0000   0.0000   0.0000
AVEPUB    0.0312   0.0000   0.0000   0.0000   0.0000
blank     0.0000   0.0000   0.0000   0.0000   0.0000
CENJAC    0.2812   0.0000   0.0000   0.8125   0.0312
DACGLO    0.0312   0.0000   0.0000   0.0000   0.0000
FESRUB    0.0645   0.0000   0.0000   0.0000   0.0000
GERPRA    0.6875   0.0000   0.0312   0.0312   0.2188
HOLLAN    0.0000   0.0000   0.0000   0.0000   0.0312
KNAARV    0.0000   0.0000   0.0000   0.0000   0.0000
LEUVUL    0.5781   0.0000   0.0000   0.0000   0.5469
PHLPRA    0.0156   0.0000   0.0000   0.0000   0.0000
PLALAN    0.4062   0.8281   0.0000   0.0000   0.0000
POAPRA    0.1562   0.0000   0.0000   0.0000   0.0000
pool      0.1644   0.8082   0.0000   0.0000   0.0000
RANACR    0.0000   0.0000   0.6875   0.0000   0.0000
```

3.3 Imputation and Log Scaling

After creating the *filterMatrix*, we impute missing values with random absolute values. The imputation values are chosen accordingly to the threshold set while peak picking (intensity threshold = 100) in the data preprocessing to assure that all imputed values stay below the measured intensities. After the missing value imputation, the *dataMatrix* is scaled to log10.

```
# ---- Impute Missing Data ----
set.seed(1891)
impnum           <- abs(rnorm(sum(NAidx), mean = 70, sd = 20))
dMnoisImp        <- XallZero
dMnoisImp[NAidx] <- impnum

# ---- Log Scale ----
dMnoisImp        <- log10(dMnoisImp)
```

dataMatrix example: *before imputation: measured intensities and zero values*

```
                                           M136T92  M137T89 M137T156
pos_005_2017_B_POAPRA_B073_a_1.C.4_01_8385    0.0000    0.000    0.000
pos_005_2017_C_LEUVUL_B048_b_1.D.7_01_8396    0.0000 7001.498    0.000
pos_005_2017_D_GERPRA_C121_b_1.F.2_01_8405    0.0000    0.000 4854.355
pos_006_2017_A_RANACR_A016_b_1.B.2_01_8402  866.3226    0.000    0.000
pos_006_2017_B_RANACR_A016_b_1.C.5_01_8407 1839.2631    0.000    0.000
```

after imputation: measured intensities and random values below threshold

```
                                           M136T92   M137T89  M137T156
pos_005_2017_B_POAPRA_B073_a_1.C.4_01_8385   88.1253   64.4568   56.1964
pos_005_2017_C_LEUVUL_B048_b_1.D.7_01_8396   55.8652 7001.4980   92.4326
pos_005_2017_D_GERPRA_C121_b_1.F.2_01_8405   79.2178   62.1435 4854.3551
pos_006_2017_A_RANACR_A016_b_1.B.2_01_8402  866.3226   88.9791   63.4586
pos_006_2017_B_RANACR_A016_b_1.C.5_01_8407 1839.2631   53.5748   61.0608
```

log scaled: measured intensities and random values are log10 scaled

```
                                           M136T92 M137T89 M137T156
pos_005_2017_B_POAPRA_B073_a_1.C.4_01_8385  1.9451  1.8093   1.7497
pos_005_2017_C_LEUVUL_B048_b_1.D.7_01_8396  1.7471  3.8452   1.9658
pos_005_2017_D_GERPRA_C121_b_1.F.2_01_8405  1.8988  1.7934   3.6861
pos_006_2017_A_RANACR_A016_b_1.B.2_01_8402  2.9377  1.9493   1.8025
pos_006_2017_B_RANACR_A016_b_1.C.5_01_8407  3.2646  1.7290   1.7858
```

4 Batch Correction

Due to a large number of samples (total: 511), we split the LC-MS measurements into 12 analytical batches. Thereby, one batch included 44 samples (randomly picked from the 511 samples), one blank and several *QC* measurements. After running the batch, the MS system was cleaned and recalibrated. Besides the intensity shifts within the batches (intra-batch) caused by the slightly unstable performance of the instrument, this procedure led to further shifts across the batches (inter-batch). These intensity shifts are corrected for by calculating the introduced variance of the *QC* measurements based on principal component analysis (PCA) calculation[7]. A more detailed explanation can also be found in the corresponding manuscript by Marr et al. [1].

4.1 Preparation

First, we calculate the optimal number of components.

```
# ---- Define QC Measurements ----
idxQC                  <- which(metaPre$class == "pool")
replicates.ind         <- matrix(-1, nrow(dMnoisImp) - length(idxQC) + 1, length(idxQC))
replicates.ind[1,]     <- idxQC
replicates.ind[-1,1] <- (1:nrow(dMnoisImp))[-idxQC]

# ---- Optimal Number of Components ----
allidbafterQC <- sapply(1:15, function(NumOfComp) {
                        sprintf("Doing Batch correction using %d number of components\n",
                                NumOfComp)

                        dMnoisImpBC <- t(RUVs(x     = t(dMnoisImp),
                                              cIdx  = 1:ncol(dMnoisImp),
                                              k     = NumOfComp,
                                              scIdx = replicates.ind,
                                              round = FALSE,
                                              isLog = TRUE)$normalizedCounts)

# ---- Metadata for Evaluation ----
                 BatchCorrMetaVar    <- data.frame(SeqNr = metaPre$injectionOrder,
                                                   Batch = metaPre$batch,
                                                   SCode = metaPre$class)

                 idx <- which(metaPre$sampleType == "pool")
                 choiceDist_postBCQC <- evaluateCorrection(X    = dMnoisImpBC[idx,],
                                                           Y    = BatchCorrMetaVar[idx,],
                                                           what = "PCA",
                                                           plot = FALSE)

                        return(choiceDist_postBCQC)
                 })
```

In order to choose the optimal number of components that we use for the correction, we compare the remaining inter-batch distances on the *QC* measurements (Figure 2).

```r
# ---- Interbatch Distances vs Number of Components ----
BCdist       <- as.data.frame(allidbafterQC)
BCdist$nComp <- 1:15

compChoice   <- ggplot (BCdist, (aes(x = BCdist$nComp, y = BCdist$allidbafterQC))) +
                geom_line(size = 1) +
                geom_point(size = 6, shape = 18) +
                labs(x = "number of components", y = "inter-batch distance") +
                theme_bw()+
                theme(axis.text        = element_text(size = 25),
                      axis.title       = element_text(size = 30, face = "bold"),
                      legend.text      = element_text(size = 30),
                      panel.background = element_rect(fill = "white"),
                      panel.grid.major = element_line(colour = "grey50")
                      )
compChoice
```

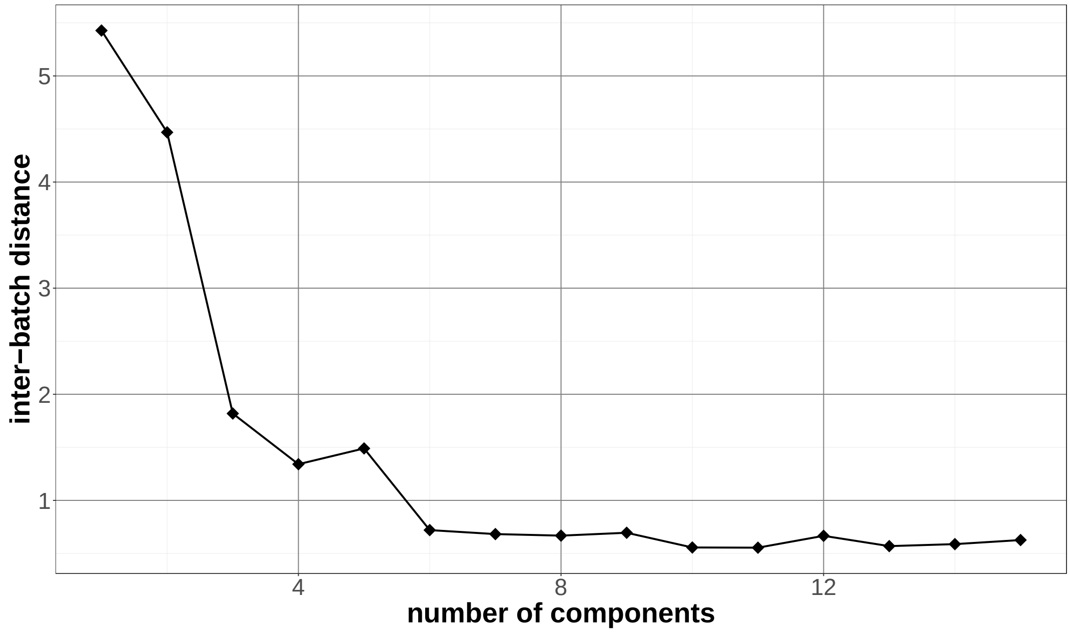

Figure 2: Number of principal components used for the batch correction keeping the inter-batch distance at a minimum.

4.2 Correction

In this example, the optimal number of components (*nComp*) is six, as the remaining inter-batch distance (*allidbafterQC*) decreases only slightly with more components.

```
print(BCdist)
```

```
   allidbafterQC nComp
1      5.4277283     1
2      4.4684877     2
3      1.8185237     3
4      1.3409498     4
5      1.4896308     5
6      0.7199777     6
7      0.6824285     7
8      0.6677630     8
9      0.6949238     9
10     0.5562601    10
11     0.5543296    11
12     0.6660080    12
13     0.5690315    13
14     0.5878562    14
15     0.6262616    15
```

Hence, we perform the batch correction with six components and save the corresponding metadata for the evaluation.

```
# ----- Batch Correction ----
NumOfComp <-  6

dMnoisImpBC <- t(RUVs(x  = t(dMnoisImp),
                 cIdx  = 1:ncol(dMnoisImp),
                 k     = NumOfComp,
                 scIdx = replicates.ind,
                 round = FALSE,
                 isLog = TRUE)$normalizedCounts)

# ---- Metadata for Evaluation ----
BatchCorrMetaVar <- data.frame(SeqNr = metaPre$injectionOrder,
                               Batch = metaPre$batch,
                               SCode = metaPre$class)
```

4.3 Evaluation

To evaluate the applied batch correction, we calculate the inter-batch distances before the correction and after the correction for all measurements (including quality controls) and the *QC* measurements separately (Figure 3).

```
# ---- Batch Correction Evaluation ----
Dist_preBCQC  <- evaluateCorrection(X    = dMnoisImp[idxQC,],
                                    Y    = BatchCorrMetaVar[idxQC,],
                                    what = "PCA",
                                    plot = FALSE)
Dist_postBCQC <- evaluateCorrection(X    = dMnoisImpBC[idxQC,],
                                    Y    = BatchCorrMetaVar[idxQC,],
                                    what = "PCA",
                                    plot = FALSE)
Dist_preBC    <- evaluateCorrection(X    = dMnoisImp,
                                    Y    = BatchCorrMetaVar,
                                    what = "PCA",
                                    plot = FALSE)
Dist_postBC   <- evaluateCorrection(X    = dMnoisImpBC,
                                    Y    = BatchCorrMetaVar,
                                    what = "PCA",
                                    plot = FALSE)
```

We used PCA to visualise the effects of the correction on all measurement and *QC* measurements separately.

```
# ---- Calculate PCA Before and After Correction: QC ----
idxQC <- which(metaPre$sampleType == "pool")

pca_Dist_preBCQC  <- FactoMineR::PCA(dMnoisImp[idxQC,],
                                     quali.sup  = w1,
                                     quanti.sup = w2,
                                     scale.unit = TRUE,
                                     ncp        = 6,
                                     graph      = FALSE)
pca_Dist_postBCQC <- FactoMineR::PCA(dMnoisImpBC[idxQC,],
                                     quali.sup  = w1,
                                     quanti.sup = w2,
                                     scale.unit = TRUE,
                                     ncp        = 6,
                                     graph      = FALSE)

# ---- Calculate PCA Before and After Correction: Samples and Quality Controls ----
pca_Dist_preBC    <- FactoMineR::PCA(dMnoisImp,
                                     quali.sup  = w1,
                                     quanti.sup = w2,
                                     scale.unit = TRUE,
                                     ncp        = 6,
                                     graph      = FALSE)
pca_Dist_postBC   <- FactoMineR::PCA(dMnoisImpBC,
                                     quali.sup  = w1,
                                     quanti.sup = w2,
                                     scale.unit = TRUE,
                                     ncp        = 6,
                                     graph      = FALSE)
```

```r
# ---- Plot PCA Before Correction: QC ----
idxQC <- which(metaPre$sampleType == "pool")

preBCQC <- fviz_pca_ind(pca_Dist_preBCQC,
                        axes        = c(1, 2),
                        geom        = c("point"),
                        addEllipses = F,
                        pointsize   = 6,
                        habillage   = metaPre[idxQC,]$batch,
                        mean.point  = FALSE,
                        title       = paste0("QC Distance before Correction: ",
                                       format(signif(Dist_preBCQC, 4), nsmall = 4))) +
           labs(x = paste0("PC 1 (",
                           format(round(pca_Dist_preBCQC$eig[1, 2], 4),
                                  nsmall = 4, digits = 4), "%)"),
                y = paste0("PC 2 (",
                           format(round(pca_Dist_preBCQC$eig[2, 2], 4),
                                  nsmall = 4, digits = 4), "%)"),
                tag = "a)") +
  scale_x_continuous(limits = c(-50, 100), breaks = seq(-100, 100, by = 40)) +
  scale_y_continuous(limits = c(-40,  40), breaks = seq(-100, 100, by = 40)) +
  scale_shape_manual(values = c(15, 16, 17, 18, 8, 25, 9, 10, 15, 16, 17, 18),
                     name   = "Batch") +
  scale_color_manual(values = c("#CC0C00FF", "#FFCD00FF", "#00AF66FF",
                                "#5C88DAFF", "#7C878EFF", "#84BD00FF",
                                "#00B5E2FF", "#CC0C00FF", "#FFCD00FF",
                                "#00AF66FF", "#5C88DAFF", "#7C878EFF"),
                     name   = "Batch") +
  theme_bw()+
    theme(axis.text         = element_text(size = 25),
        axis.title          = element_text(size = 30, face = "bold"),
        panel.background    = element_rect(fill = "white"),
        panel.grid.major    = element_line(colour = "grey50"),
        plot.tag            = element_text(size = 30),
        plot.title          = element_text(size = 25),
        legend.position     = "none",
        legend.title        = element_text(size = 30),
        legend.text         = element_text(size = 30)
        )
```

```r
# ---- Plot PCA After Correction: QC ----
postBCQC <- fviz_pca_ind(pca_Dist_postBCQC,
                         axes        = c(1, 2),
                         geom        = c("point"),
                         addEllipses = F,
                         pointsize   = 6,
                         habillage   = metaPre[idxQC, ]$batch,
                         mean.point  = FALSE,
                         title       = paste0("QC Distance after Correction: ",
                                              format(Dist_postBCQC, nsmall = 4, digits = 4))) +
            labs(x = paste0("PC 1 (",
                            format(round(pca_Dist_postBCQC$eig[1, 2], 4),
                                   nsmall = 4, digits = 4), "%)"),
                 y = paste0("PC 2 (",
                            format(round(pca_Dist_postBCQC$eig[2, 2], 4),
                                   nsmall = 4, digits = 4), "%)"),
                 tag  = "b)",
                 fill = "Batch") +
            scale_x_continuous(limits = c(-50, 100), breaks = seq(-100, 100, by = 40)) +
            scale_y_continuous(limits = c(-40,  40), breaks = seq(-100, 100, by = 40)) +
            scale_shape_manual(values = c(15, 16, 17, 18, 8, 25, 9, 10, 15, 16, 17, 18),
                               name   = "Batch") +
            scale_color_manual(values = c("#CC0C00FF", "#FFCD00FF", "#00AF66FF",
                                          "#5C88DAFF", "#7C878EFF", "#84BD00FF",
                                          "#00B5E2FF", "#CC0C00FF", "#FFCD00FF",
                                          "#00AF66FF", "#5C88DAFF", "#7C878EFF"),
                               name   = "Batch") +
            theme_bw() +
            theme(axis.text             = element_text(size = 25),
                  axis.title            = element_text(size = 30, face = "bold"),
                  panel.background      = element_rect(fill = "white"),
                  panel.grid.major      = element_line(colour = "grey50"),
                  plot.tag              = element_text(size = 30),
                  plot.title            = element_text(size = 25),
                  legend.direction      = "vertical",
                  legend.justification = c("top"),
                  legend.margin         = margin(8, 8, 8, 8),
                  legend.position       = c(0.85, 0.90),
                  legend.title          = element_text(size = 30),
                  legend.text           = element_text(size = 25)
                  )
```

```r
# ---- Plot PCA Before Correction: Samples and Quality Controls ----
preBC <- fviz_pca_ind(pca_Dist_preBC,
                      axes        = c(1, 2),
                      geom        = c("point"),
                      addEllipses = FALSE,
                      habillage   = metaPre$batch,
                      mean.point  = FALSE,
                      pointsize   = 6,
                      title       = paste0("Distance before Correction: ",
                                      format(round(Dist_preBC, 4),
                                             digits = 4, nsmall = 4))) +
         labs(x = paste0("PC 1 (",
                         format(round(pca_Dist_preBC$eig[1, 2], 4),
                                nsmall = 4, digits = 4),
                         "%)"),
              y = paste0("PC 2 (",
                         format(round(pca_Dist_preBC$eig[2, 2], 4),
                                nsmall = 4, digits = 4),
                         "%)"),
              tag = "c)") +
         scale_x_continuous(limits = c(-50, 100), breaks = seq(-100, 100, by = 40)) +
         scale_y_continuous(limits = c(-60,  60), breaks = seq(-100, 100, by = 40)) +
         scale_shape_manual(values = c(15, 16, 17, 18, 8, 25, 9, 10, 15, 16, 17, 18),
                            name   = "Batch") +
         scale_color_manual(values = c("#CC0C00FF", "#FFCD00FF", "#00AF66FF",
                                       "#5C88DAFF", "#7C878EFF", "#84BD00FF",
                                       "#00B5E2FF", "#CC0C00FF", "#FFCD00FF",
                                       "#00AF66FF", "#5C88DAFF", "#7C878EFF"),
                            name   = "Batch") +
         theme_bw() +
         theme(axis.text        = element_text(size = 25),
               axis.title       = element_text(size = 30, face = "bold"),
               panel.background = element_rect(fill = "white"),
               panel.grid.major = element_line(colour = "grey50"),
               plot.tag         = element_text(size = 30),
               plot.title       = element_text(size = 25),
               legend.position  = "none",
               legend.title     = element_text(size = 30),
               legend.text      = element_text(size = 30)
               )
```

```r
# ---- Plot PCA After Correction: Samples and Quality Controls ----
postBC <- fviz_pca_ind(pca_Dist_postBC,
                       axes        = c(1, 2),
                       addEllipses = F,
                       geom        = c("point"),
                       pointsize   = 6,
                       habillage   = metaPre$batch,
                       mean.point  = FALSE,
                       title       = paste0("Distance after Correction: ",
                                            format(round(Dist_postBC, 4),
                                                   nsmall = 4, digits = 4))) +
          labs(x = paste0("PC 1 (",
                          format(round(pca_Dist_postBC$eig[1, 2], 4),
                                 nsmall = 4, digits = 4),
                          "%)"),
               y = paste0("PC 2 (",
                          format(round(pca_Dist_postBC$eig[2, 2], 4),
                                 nsmall = 4, digits = 4),
                          "%)"),
               tag = "d)") +
          scale_x_continuous(limits = c(-50, 100), breaks = seq(-100, 100, by = 40)) +
          scale_y_continuous(limits = c(-60,  60), breaks = seq(-100, 100, by = 40)) +
          scale_shape_manual(values = c(15, 16, 17, 18, 8, 25, 9, 10, 15, 16, 17, 18),
                             name   = "Batch") +
          scale_color_manual(values = c("#CC0C00FF", "#FFCD00FF", "#00AF66FF",
                                        "#5C88DAFF", "#7C878EFF", "#84BD00FF",
                                        "#00B5E2FF", "#CC0C00FF", "#FFCD00FF",
                                        "#00AF66FF", "#5C88DAFF", "#7C878EFF"),
                             name   = "Batch") +
          theme_bw() +
          theme(axis.text        = element_text(size = 25),
                axis.title       = element_text(size = 30, face = "bold"),
                panel.background = element_rect(fill = "white"),
                panel.grid.major = element_line(colour = "grey50"),
                plot.tag         = element_text(size = 30),
                plot.title       = element_text(size = 25),
                legend.position  = "none",
                legend.title     = element_text(size = 30),
                legend.text      = element_text(size = 30)
                )

# ---- Arrange Batch Correction Plots ----
# grid.arrange(preBC, postBC, preBCQC, postBCQC, nrow = 2, ncol = 2)
```

The inter-batch distances in the *QC* measurements before (16.8450445) and after (0.7199777) the batch correction show that the data set is subject to unwanted variations. However, looking at all measurements before (0.056425) and after (0.057719) the correction, the inter-batch distances do not change significantly, revealing that the variation between the samples is much higher than the variation in the *QC* measurements that were introduced by the batches. PCA plots visualise the effects of batch correction on both *QC* measurements and analytical samples.

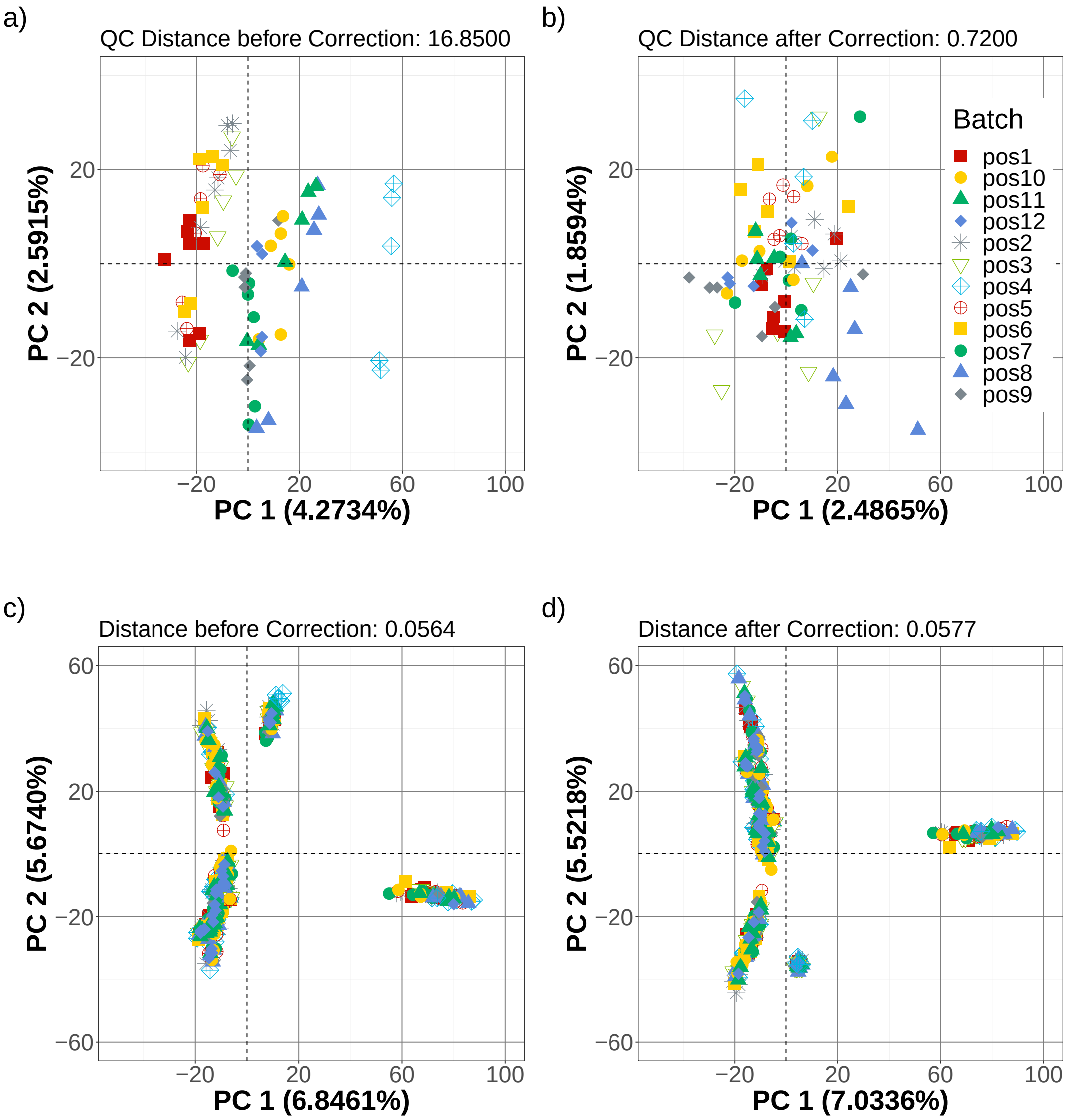

Figure 3: Inter-batch distances before and after batch correction. Distances are calculated across 12 analytical LC-MS batches. Distances before and after correction are compared for the *QC* measurements and for the sample measurements, respectively. The *QC* measurements show a high inter-batch distance (a) that is reduced significantly after correction (b) , while the distances detected for the analytical samples (c) only change slightly after correction (d). The proportion of the total explained variance is shown for each component in brackets.

5 Validity Check

5.1 Feature Validity

Blank Removal

All features were checked for their validity. We only kept those features in the *dataMatrix* that were not detected in the blanks, as these features are likely to have been introduced unintendedly through extraction or while measurements. Therefore, all features detected in blanks were removed from the *dataMatrix* and excluded from further analysis.

```
# ---- Keep Processed Data ----
dMnoisImpSBC  <- dMnoisImpBC
dMnoisImpS    <- dMnoisImp
meta          <- metaPre
NAidxS        <- NAidx

# ---- Remove Blank-Features ----
mzrtnames     <- names(which(filterMatrix["blank", ] >0))
tempColnames  <- setdiff(colnames(dMnoisImpSBC), mzrtnames)
NAidxS        <- NAidxS[ , tempColnames]
filterMatrixS <- filterMatrix[ , tempColnames]
dMnoisImpSBC  <- dMnoisImpSBC[ , tempColnames]
dMnoisImpS    <- dMnoisImpS[ ,  tempColnames]
```

QC Removal

In this example, we used the quality controls *QC* and blanks for batch correction and feature validity checks, respectively. These quality controls are not needed for further analysis and are, therefore, removed from both the *dataMatrix* and *sampleMetadata*.

```
# ---- Remove QC Measurements ----
idxQC         <- meta$SpecCode != "pool"

meta          <- meta[idxQC, ]
NAidxS        <- NAidxS[idxQC, ]
dMnoisImpSBC  <- dMnoisImpSBC[idxQC, ]
dMnoisImpS    <- dMnoisImpS[idxQC, ]

# ---- Remove Blanks ----
idxB          <- meta$SpecCode != "blank"
meta          <- meta[idxB, ]
NAidxS        <- NAidxS[idxB, ]
dMnoisImpSBC  <- dMnoisImpSBC[idxB, ]
dMnoisImpS    <- dMnoisImpS[idxB, ]

# ---- Drop Unused Levels ----
meta[ , w1]    <- data.frame(lapply(w1, function(ii){
                    base::droplevels(meta[ , ii])
                  }))
```

5.2 Sample Validity

As the last step of processing, we also checked the analytical samples for their validity. In our example, the data set is derived from a field experiment that is incorporating a high level of variance on both the inter-species and intra-species level. We, therefore, test all samples within their respective species for feature composition similarity, ensuring they are not contaminated or corrupted. A sample is considered valid when its Mahalanobis distance to its species cluster does not exceed the distance of the 3-fold mean (Figure 4) and when it shares at least 0.25 of features with the other samples in its respective species. Therefore, we calculate the Mahalanobis distances for all species subsets separately.

```
# ---- Mahalanobis Distances ----
lbls             <- levels(meta$SpecCode)
OutlierDist_list <- list()

for (lbl in lbls) {
  idxSpec             <- meta$SpecCode == lbl
  zeroBCdata          <- dMnoisImpSBC
  zeroBCdata[NAidxS]  <- 0
  idxF                <- colSums(zeroBCdata[idxSpec, ], na.rm = TRUE) != 0
  sdat                <- zeroBCdata[idxSpec, idxF]
  idxFS               <- which( apply(sdat, 2,
                                  function(i) { sum( i != 0) /length(i)}) >= 0.25)
  sdatMin             <- sdat[, idxFS]
  fShared             <- specnumber(sdatMin)/ncol(sdatMin)
  share               <- 0.25
  idxfShared          <- fShared < share
  pcamdl              <- prcomp(sdat, center = TRUE, scale.= TRUE)
  sdat                <- pcamdl$x[ , 1:20]
  myLOOMDdist         <- sapply(1:nrow(sdat), function(i) {
                            mahalanobis(sdat[i, ], colMeans(sdat[-i, ], na.rm = TRUE),
                                        cov(sdat[-i, ]))
                          })
  maxDist             <- 3 * mean(myLOOMDdist)
  idxLOOMD            <- myLOOMDdist > maxDist


  OD                  <- matrix(myLOOMDdist,
                               nrow = length(myLOOMDdist),1,
                               dimnames = list(rownames(sdat), c("Mdist")))
  OutlierDist         <- merge(sdat, OD, by = "row.names")

  OutlierDist_list[[lbl]] <- OutlierDist

}

OutlierDist_all  <- do.call(rbind, OutlierDist_list)
```

We plot all samples indicating the distance to their respective species.

```
# ---- Set Variables ----
SampleNames <- as.factor(OutlierDist_all$Row.names)
maxDist       <- 3 * mean(OutlierDist_all$Mdist)
```

```r
# ---- Plot Mahalanobis Distances for All Samples ----
MD_allSamples <- ggplot(data       = OutlierDist_all,
                        aes(x      = SampleNames,
                            y      = Mdist, #OutlierDist_all$Mdist,
                            colour = meta$SpecCode,
                            shape  = meta$SpecCode
                            )) +
                 geom_point(size = 6) +
                 labs(x = paste0("samples"),
                      y = paste0("mahalanobis distance")) +
                 theme_bw() +
                 theme(axis.text        = element_text(size = 25),
                       axis.title       = element_text(size = 30, face = "bold"),
                       axis.text.x      = element_blank(),
                       axis.ticks.x     = element_blank(),
                       legend.text      = element_markdown(size = 30),
                       legend.position  = "right",
                       legend.title     = element_text(size = 30),
                       panel.background = element_rect(fill = "white"),
                       panel.grid.major = element_blank(),
                       panel.grid       = element_blank()
                       ) +
                 scale_color_manual(name   = "Species",
                                   values = c("#DC0000FF", "#7E6148FF", "#3C5488FF",
                                              "#3C5488FF", "#F39B7FFF", "#4DBBD5FF",
                                              "#8491B4FF", "#8491B4FF", "#F39B7FFF",
                                              "#91D1C2FF", "#00A087FF", "#B09C85FF",
                                              "#E64B35FF"),
                                   labels = c("*Anthoxanthum odoratum*",
                                              "*Avenula pubescens*",
                                              "*Centaurea jacea*",
                                              "*Dactylis glomerata*",
                                              "*Festuca rubra*",
                                              "*Geranium pratense*",
                                              "*Holcus lanatus*",
                                              "*Knautia arvensis*",
                                              "*Leucanthemum vulgare*",
                                              "*Phleum pratense*",
                                              "*Plantago lanceolata*",
                                              "*Poa pratensis*",
                                              "*Ranunculus acris*")) +
                 scale_shape_manual(name   = "Species",
                                   values = c(8, 10, 16, 24, 8, 9, 17,
                                              25, 18, 15, 18, 15, 9),
                                   labels = c("*Anthoxanthum odoratum*",
                                              "*Avenula pubescens*",
                                              "*Centaurea jacea*",
                                              "*Dactylis glomerata*",
                                              "*Festuca rubra*",
                                              "*Geranium pratense*",
                                              "*Holcus lanatus*",
                                              "*Knautia arvensis*",
                                              "*Leucanthemum vulgare*",
                                              "*Phleum pratense*",
                                              "*Plantago lanceolata*",
                                              "*Poa pratensis*",
                                              "*Ranunculus acris*")) +
                 geom_abline(intercept = maxDist, color = "red", size = 1.5)

MD_allSamples
```

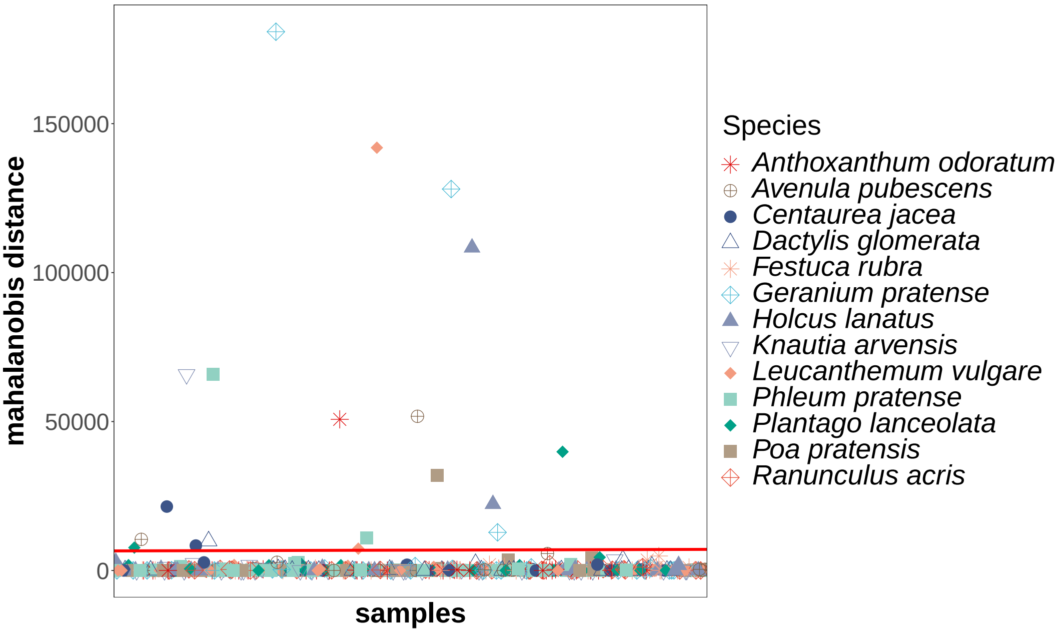

Figure 4: Sample validity check. Calculating the Mahalanobis distances for each species separately. Thresholds (3-fold mean distance within species) are calculated species specific. The indicated threshold (red line) representes the 3-fold distance of the total mean and is included for demonstration purposes only. Only samples above their respective threshold and < 0.25 shared features with their respective species are excluded from further analysis.

Mahalanobis distances and the corresponding feature composition is calculated for all samples within their species separately.

```
# ---- Species Specific Validity Check ----
metaOut   <- meta
corrSamp  <- unlist(sapply(levels(meta$SpecCode), function(lbl) {
  idxSpec            <- meta$SpecCode == lbl

  zeroBCdata         <- dMnoisImpSBC
  zeroBCdata[NAidxS] <- 0


  # ---- Species Subset ----
  idxF        <- colSums(zeroBCdata[idxSpec,], na.rm = TRUE)!= 0
  sdat        <- zeroBCdata[idxSpec, idxF]

  idxFS       <- which( apply(sdat, 2, function(i) { sum( i != 0) /length(i)}) >= 0.25)
  sdatMin     <- sdat[, idxFS]

  # ---- Feature Composition ----
  fShared     <- specnumber(sdatMin)/ncol(sdatMin)
  share       <- 0.25
  idxfShared  <- fShared < share

  pcamdl      <- prcomp(sdat, center = TRUE, scale.=TRUE)
  sdat        <- pcamdl$x[,1:20]

  # ---- Mahalanobis Distances ----
  myLOOMDdist <- sapply(1:nrow(sdat), function(i) {
                   mahalanobis(sdat[i,], colMeans(sdat[-i,], na.rm = TRUE),  cov(sdat[-i,]))
  })

  # ---- Threshold Distance ----
  maxDist     <- 3 * mean(myLOOMDdist)
  idxLOOMD    <- myLOOMDdist > maxDist

  return(rownames(sdat)[idxLOOMD & idxfShared])

}))
```

In this data set, 12 samples did not pass the validity check. We, therefore, excluded them from the *dataMatrix* and further analysis.

```
 [1] "pos_066_2017_C_ANTODO_B060_b_1.A.6_01_9086"
 [2] "pos_078_2017_B_ANTODO_A018_b_1.A.5_01_9085"
 [3] "pos_036_2017_A_HOLLAN_A035_b_2.A.7_01_9037"
 [4] "pos_110_2017_D_LEUVUL_B051_a_1.F.8_01_8877"
 [5] "pos_116_2017_D_LEUVUL_B051_b_1.F.3_01_8966"
 [6] "pos_018_2017_D_PHLPRA_B073_b_2.F.4_01_8481"
 [7] "pos_049_2017_D_PHLPRA_A018_b_1.F.2_01_8529"
 [8] "pos_053_2017_D_PHLPRA_B059_b_1.F.6_01_8559"
 [9] "pos_070_2017_D_PHLPRA_A018_a_1.F.1_01_8688"
[10] "pos_016_2017_D_POAPRA_A026_b_2.F.2_01_8484"
[11] "pos_057_2017_D_POAPRA_B073_a_2.E.7_01_8626"
[12] "pos_073_2017_D_POAPRA_B073_b_1.F.4_01_8682"
```

```
# ---- Remove Samples ----
idxVfyd         <- which(!(rownames(dMnoisImpSBC) %in% corrSamp))
dMnoisImpSBCOut <- dMnoisImpSBC[idxVfyd, ]
metaOut         <- metaOut[idxVfyd, ]
```

6 Processed Data Output

6.1 Data Saving

After performing the validity checks on the data, we save all processed data matrices.

```
# ---- Save processed data matrices ----
prcdSampleMetadata <- metaOut
prcdDataMatrixImputedLog <- dMnoisImpSBCOut
prcdDataMatrixImputed <- 10^prcdDataMatrixImputedLog

prcdDataMatrixZero <- prcdDataMatrixImputed
NAidxPrcd <- NAidxS[rownames(prcdDataMatrixImputed), colnames(prcdDataMatrixImputed)]
prcdDataMatrixZero[NAidxPrcd] <- 0


# combined meta data and data matrix
prcdMetadataMatrix <- cbind(prcdSampleMetadata, prcdDataMatrixZero, make.row.names = TRUE)
```

6.2 Filter Matrix Re-calculation

In order to apply further statistics to the data, we also need to recalculate the *filterMatrix* with the processed data matrix (*prcdDataMatrixZero*) and the corresponding sample metadata (*prcdSampleMetadata*).

	M127T743	M131T466	M132T149	M133T212	M133T467
ANTODO	0.0333	0.0000	0.0000	0.0000	0.0000
AVEPUB	0.0312	0.0000	0.0000	0.0000	0.0000
CENJAC	0.2812	0.0000	0.0000	0.8125	0.0312
DACGLO	0.0312	0.0000	0.0000	0.0000	0.0000
FESRUB	0.0645	0.0000	0.0000	0.0000	0.0000
GERPRA	0.6875	0.0000	0.0312	0.0312	0.2188
HOLLAN	0.0000	0.0000	0.0000	0.0000	0.0323
KNAARV	0.0000	0.0000	0.0000	0.0000	0.0000
LEUVUL	0.5968	0.0000	0.0000	0.0000	0.5645
PHLPRA	0.0167	0.0000	0.0000	0.0000	0.0000
PLALAN	0.4062	0.8281	0.0000	0.0000	0.0000
POAPRA	0.1724	0.0000	0.0000	0.0000	0.0000
RANACR	0.0000	0.0000	0.6875	0.0000	0.0000

7 Visualisation

Here, we use PCA to visualise the processed data. The functional-group-wise calculation shows good species separation for both *grass* and *herb* species (Figure 5). For a more detailed explanation see also the corresponding manuscript by Marr et al. [1]

```
# ---- Calculate PCA for All Samples ----
pcaGlobal <- FactoMineR::PCA(dMnoisImpSBCOut,
                             quali.sup   = w1,
                             quanti.sup = w2,
                             scale.unit = TRUE,
                             ncp        = 6,
                             graph      = F)

# ---- Calculate PCA Functional Group Specific: Grass ----
idxGrass <- subset(dMnoisImpSBCOut,
                   metaOut$FuncGroup == "grass")
pcaGrass <- FactoMineR::PCA(idxGrass,
```

```
                             quali.sup   = w1,
                             quanti.sup = w2,
                             scale.unit = TRUE,
                             ncp         = 6,
                             graph       = F)

# ---- Calculate PCA Functional Group Specific: Herb ----
idxHerb <- subset(dMnoisImpSBCOut,
                  metaOut$FuncGroup == "herb")
pcaHerb <- FactoMineR::PCA(idxHerb,
                            quali.sup    = w1,
                            quanti.sup   = w2,
                            scale.unit   = TRUE,
                            ncp          = 6,
                            graph        = F)
```

```
# ---- Plot All Samples ----
pcaGlobalplot <- fviz_pca_ind(pcaGlobal,
                              axes        = c(1,2),
                              addEllipses = FALSE,
                              geom        = c("point"),
                              pointsize   = 6,
                              habillage   = metaOut$SpecCode,
                              mean.point  = FALSE,
                              title       = paste0("Functional Group: Grass & Herb")) +
                 labs(x   = paste0("PC 1 (",
                                   format(round(pcaGlobal$eig[1,2], 4),
                                          nsmall = 4, digits = 4), "%)"),
                      y   = paste0("PC 2 (",
                                   format(round(pcaGlobal$eig[2,2], 4),
                                          nsmall = 4, digits = 4), "%)"),
                      tag = "a)") +
                 theme_bw() +
                 theme(axis.text        = element_text(size = 25),
                       axis.title       = element_text(size = 30, face = "bold"),
                       legend.position  = "bottom",
                       legend.text      = element_text(size = 30),
                       legend.title     = element_text(size = 30, colour = "white"),
                       panel.background = element_rect(fill = "white"),
                       panel.grid.major = element_line(colour = "grey50"),
                       plot.tag         = element_text(size = 30),
                       plot.title       = element_text(size = 25)
                       ) +
                 scale_x_continuous(limits = c(-80,  90), breaks = seq(-100, 100, by = 40)) +
                 scale_y_continuous(limits = c(-50, 100), breaks = seq(-100, 100, by = 40)) +
                 scale_shape_manual(values = c(8, 10, 16, 24, 8, 9, 17,
                                               25, 18, 15, 18, 15, 9)) +
                 scale_color_manual(values = c("#DC0000FF", "#7E6148FF", "#3C5488FF",
                                               "#3C5488FF", "#F39B7FFF", "#4DBBD5FF",
                                               "#8491B4FF", "#8491B4FF", "#F39B7FFF",
                                               "#91D1C2FF", "#00A087FF", "#B09C85FF",
                                               "#E64B35FF"))
```

```r
# ---- Plot Functional Group Specific: Grass ----
idxGrass     <- subset(dMnoisImpSBCOut, metaOut$FuncGroup == "grass")
idxGrassMeta <- subset(metaOut, metaOut$FuncGroup == "grass")

pcaGrassplot <- fviz_pca_ind(pcaGrass,
                             axes        = c(1,2),
                             addEllipses = FALSE,
                             geom        = c("point"),
                             pointsize   = 6,
                             habillage   = idxGrassMeta$SpecCode,
                             mean.point  = FALSE,
                             title       = paste0("Functional Group: Grass")) +
                labs(x   = paste0("PC 1 (",
                                  format(round(pcaGrass$eig[1,2], 4), nsmall = 4, digits = 4),
                                  "%)"),
                     y   = paste0("PC 2 (",
                                  format(round(pcaGrass$eig[2,2], 4), nsmall = 4, digits = 4),
                                  "%)"),
                     tag = "b)") +
                theme_bw()+
                theme(axis.text        = element_text(size = 25),
                      axis.title       = element_text(size = 30, face = "bold"),
                      legend.position  = "none",
                      panel.background = element_rect(fill = "white"),
                      panel.grid.major = element_line(colour = "grey50"),
                      plot.tag         = element_text(size = 30),
                      plot.title       = element_text(size = 25)
                      ) +
                scale_x_continuous(limits = c(-80,  90), breaks = seq(-100, 100, by = 40)) +
                scale_y_continuous(limits = c(-50, 100), breaks = seq(-100, 100, by = 40)) +
                scale_shape_manual(values = c(8, 10, 24, 8, 17, 15, 15)) +
                scale_color_manual(values = c("#DC0000FF", "#7E6148FF", "#3C5488FF",
                                              "#F39B7FFF", "#8491B4FF", "#91D1C2FF",
                                              "#B09C85FF"))
```

```r
# ---- Plot Functional Group Specific: Herb ----
idxHerb     <- subset(dMnoisImpSBCOut, metaOut$FuncGroup == "herb")
idxHerbMeta <- subset(metaOut, metaOut$FuncGroup == "herb")

pcaHerbplot <- fviz_pca_ind(pcaHerb,
                            axes        = c(1,2),
                            addEllipses = FALSE,
                            geom        = c("point"),
                            pointsize   = 6,
                            habillage   = idxHerbMeta$SpecCode,
                            mean.point  = FALSE,
                            title       = paste0("Functional Group: Herb")) +
              labs(x   = paste0("PC 1 (",
                              format(round(pcaHerb$eig[1,2], 4), nsmall = 4, digits = 4),
                              "%)"),
                   y   = paste0("PC 2 (",
                              format(round(pcaHerb$eig[2,2], 4), nsmall = 4, digits = 4),
                              "%)"),
                   tag = "c)") +
              theme_bw()+
              theme(axis.text        = element_text(size = 25),
                    axis.title       = element_text(size = 30, face = "bold"),
                    legend.position  = "none",
                    panel.background = element_rect(fill = "white"),
                    panel.grid.major = element_line(colour = "grey50"),
                    plot.tag         = element_text(size = 30),
                    plot.title       = element_text(size = 25)
                    ) +
              scale_x_continuous(limits = c(-80,  90), breaks = seq(-100, 100, by = 40)) +
              scale_y_continuous(limits = c(-50, 100), breaks = seq(-100, 100, by = 40)) +
              scale_shape_manual(values = c(16, 9, 25, 18, 18, 9)) +
              scale_color_manual(values = c("#3C5488FF", "#4DBBD5FF", "#8491B4FF",
                                            "#F39B7FFF", "#00A087FF", "#E64B35FF"))

# ---- Arrange PCA Plots ----

# pL <- list(pcaGlobalplot, pcaGrassplot, pcaHerbplot)
# grid.arrange(
#    grobs = pL,
#    widths = c(2, 2, 1),
#    layout_matrix = rbind(c(1, 1),
#                          c(2, 3))
# )
```

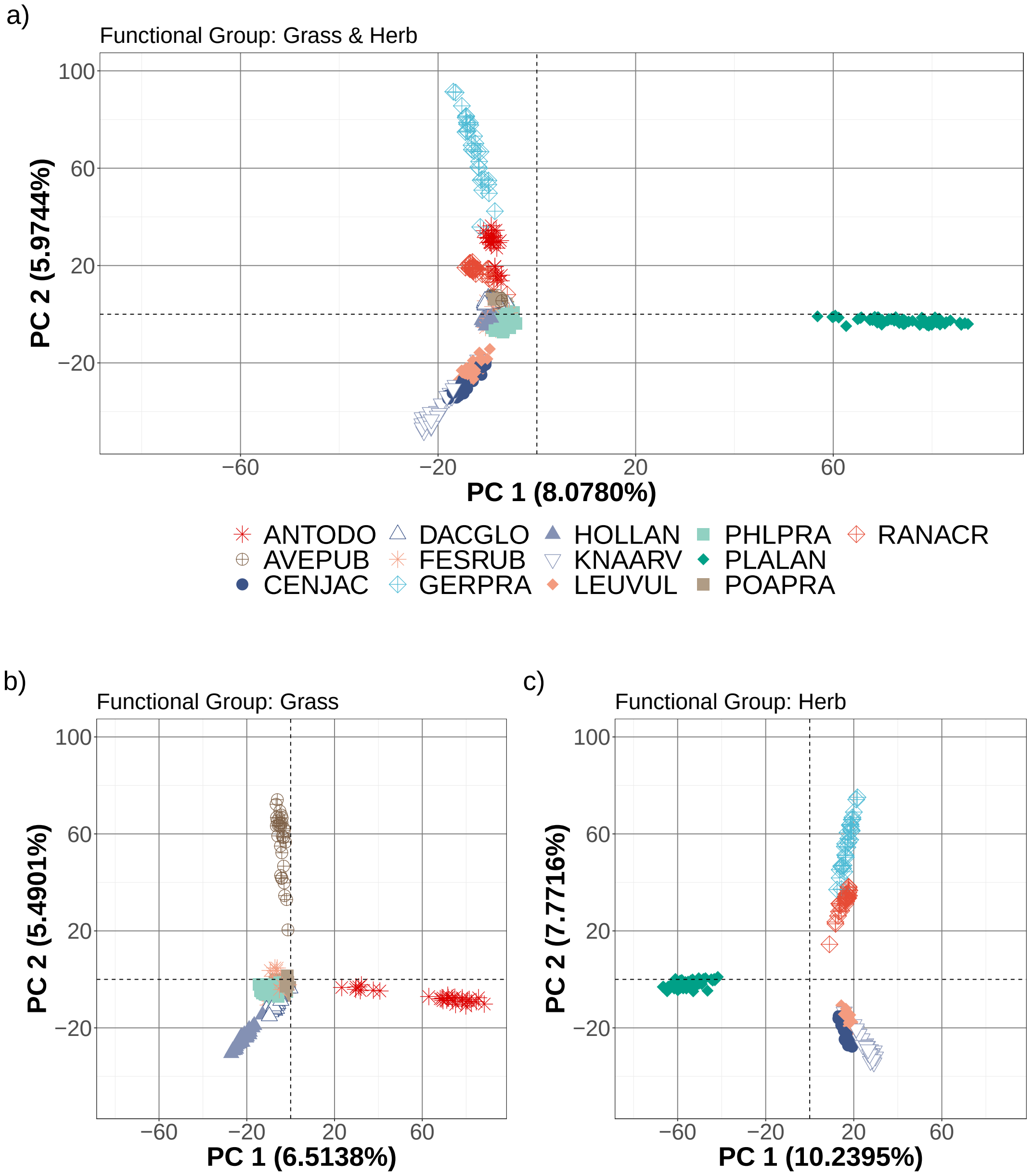

Figure 5: Sample selection for statistical analysis. The separation of the samples by principle components shows that samples belonging to the same species are grouped in clusters (a). Samples separated by functional groups *grass* (b) and *herb* (c), are still found to group in their respective species clusters. We used species abbreviations in the plot's key for clarity reasons: *Anthoxanthum odoratum* (ANTODO), *Avenula pubescens* (AVEPUB), *Centaurea jacea* (CENJAC), *Dactylis glomerata* (DACGLO), *Festuca rubra* (FESRUB), *Geranium pratense* (GERPRA), *Holcus lanatus* (HOLLAN), *Knautia arvensis* (KNAARV), *Leucanthemum vulgare* (LEUVUL), *Phleum pratense* (PHLPRA), *Plantago lanceolata* (PLALAN), *Poa pratensis* (POAPRA), *Ranunculus acris* (RANACR).

8 References

1 Marr, S., Hageman, J. A., Wehrens, R., van Dam, N. M., Bruelheide, H. & Neumann, S. LC-MS based plant metabolic profiles of thirteen grassland species grown in diverse neighbourhoods. *Sci. Data* (supplemental material).

2 Giacomoni, F., Le Corguillé, G., Monsoor, M., Landi, M., Pericard, P., Pétéra, M., Duperier, C., Tremblay-Franco, M., Martin, J.-F., Jacob, D., Goulitquer, S., Thévenot, E. A. & Caron, C. Workflow4Metabolomics: a collaborative research infrastructure for computational metabolomics. *Bioinformatics* **31** (9), 1493-1495 (2014).

3 Allaire, J. J., Xie, Y., McPherson, J., Luraschi, J., Ushey, K., Atkins, A., Wickham, H., Cheng, J., Chang, W. & Iannone, R. rmarkdown: Dynamic Documents for R. R package version 2.0. URL https://rmarkdown.rstudio.com (2019).

4 Xie, Y., Allaire, J. J. & Grolemund, G. R Markdown: The Definitive Guide. URL https://bookdown.org/yihui/rmarkdown (Chapman and Hall, 2018).

5 Wehrens, R., Mumm, R., Keurentjes, J. & de Vos, R. BatchCorrMetabolomics: Complementary package to "Improved Batch Correction in Untargeted MS-Based Metabolomics". R package version 0.1.14. (2020).

6 Bache, S. M. & Wickham, H. magrittr: A Forward-Pipe Operator for R. R package version 1.5. https://CRAN.R-project.org/package=magrittr (2014).

7 Risso, D., Ngai, J., Speed, T. P. & Dudoit, S. Normalization of RNA-seq data using factor analysis of control genes or samples. *Nat. Biotechnol.* **32** (9), 896-902 (2014).

8 Brunius, C. Nifty Tools for Random Statistical Tasks. R package version 0.0.914. (2020).

9 Oksanen, J., Guillaume Blanchet, F. , Friendly, M., Kindt, R., Legendre, P., McGlinn, D., Minchin, P. R., O'Hara, R. B., Simpson, G. L., Solymos, P., Stevens, M. H. H., Szoecs, E. & Wagner, H. vegan: Community Ecology Package. R package version 2.5-6. https://CRAN.R-project.org/package=vegan (2019).

10 Kassambara, A. & Mundt, F. factoextra: Extract and Visualize the Results of Multivariate Data Analyses. R package version 1.0.6. https://CRAN.R-project.org/package=factoextra (2019).

11 Le, S., Josse, J. & Husson, F. FactoMineR: An R Package for Multivariate Analysis. *J. Stat. Softw.* **25** (1), 1-18 (2008).

12 Xiao, N. ggsci: Scientific Journal and Sci-Fi Themed Color Palettes for 'ggplot2'. R package version 2.9. https://CRAN.R-project.org/package=ggsci (2018).

13 Wilke, C. O. ggtext: Improved text rendering support for 'ggplot2'. R package version 0.1.0. https://wilkelab.org/ggtext (2020).

14 Auguie, B. gridExtra: Miscellaneous Functions for "Grid" Graphics. R package version 2.3. https://CRAN.R-project.org/package=gridExtra (2017).

15 Microsoft Corporation & Weston, S. doParallel: Foreach Parallel Adaptor for the 'parallel' Package. R package version 1.0.15. https://CRAN.R-project.org/package=doParallel (2019).

Session Info:

```
R version 4.0.3 (2020-10-10)
Platform: x86_64-w64-mingw32/x64 (64-bit)
Running under: Windows 10 x64 (build 18363)

Matrix products: default

locale:
[1] LC_COLLATE=English_World.1252      LC_CTYPE=English_World.1252
[3] LC_MONETARY=English_World.1252     LC_NUMERIC=C
[5] LC_TIME=English_United States.1252

attached base packages:
[1] stats4    parallel  stats     graphics  grDevices utils     datasets
[8] methods   base

other attached packages:
 [1] doParallel_1.0.16           iterators_1.0.13
 [3] foreach_1.5.1               ggtext_0.1.0
 [5] ggsci_2.9                   gridExtra_2.3
 [7] factoextra_1.0.7            ggplot2_3.3.2
 [9] FactoMineR_2.3              vegan_2.5-7
[11] lattice_0.20-41             permute_0.9-5
[13] StatTools_0.0.915           RUVSeq_1.24.0
[15] edgeR_3.32.0                limma_3.46.0
[17] EDASeq_2.24.0               ShortRead_1.48.0
[19] GenomicAlignments_1.26.0    SummarizedExperiment_1.20.0
[21] MatrixGenerics_1.2.0        matrixStats_0.57.0
[23] Rsamtools_2.6.0             GenomicRanges_1.42.0
[25] GenomeInfoDb_1.26.1         Biostrings_2.58.0
[27] XVector_0.30.0              IRanges_2.24.0
[29] S4Vectors_0.28.0            BiocParallel_1.24.1
[31] Biobase_2.50.0              BiocGenerics_0.36.0
[33] magrittr_2.0.1              BatchCorrMetabolomics_0.1.14
[35] knitr_1.30

loaded via a namespace (and not attached):
  [1] readxl_1.3.1           backports_1.2.0        aroma.light_3.20.0
  [4] BiocFileCache_1.14.0   splines_4.0.3          digest_0.6.27
  [7] htmltools_0.5.0        memoise_1.1.0          cluster_2.1.0
 [10] openxlsx_4.2.3         R.utils_2.10.1         sandwich_3.0-0
 [13] askpass_1.1            prettyunits_1.1.1      jpeg_0.1-8.1
 [16] colorspace_2.0-0       blob_1.2.1             rappdirs_0.3.1
 [19] ggrepel_0.8.2          haven_2.3.1            xfun_0.19
 [22] dplyr_1.0.2            crayon_1.3.4           RCurl_1.98-1.2
 [25] kohonen_3.0.10         zoo_1.8-8              glue_1.4.2
 [28] gtable_0.3.0           zlibbioc_1.36.0        DelayedArray_0.16.0
 [31] car_3.0-10             kernlab_0.9-29         prabclus_2.3-2
 [34] DEoptimR_1.0-8         abind_1.4-5            scales_1.1.1
 [37] DBI_1.1.0              rstatix_0.6.0          Rcpp_1.0.5
 [40] progress_1.2.2         gridtext_0.1.3         crch_1.0-4
 [43] scoringRules_1.0.1     foreign_0.8-80         flashClust_1.01-2
 [46] bit_4.0.4              mclust_5.4.7           Formula_1.2-4
 [49] httr_1.4.2             RColorBrewer_1.1-2     fpc_2.2-8
 [52] modeltools_0.2-23      ellipsis_0.3.1         pkgconfig_2.0.3
 [55] XML_3.99-0.5           R.methodsS3_1.8.1      flexmix_2.3-17
 [58] farver_2.0.3           nnet_7.3-14            dbplyr_2.0.0
 [61] locfit_1.5-9.4         tidyselect_1.1.0       labeling_0.4.2
 [64] rlang_0.4.9            AnnotationDbi_1.52.0   cellranger_1.1.0
 [67] munsell_0.5.0          tools_4.0.3            generics_0.1.0
```

```
 [70] RSQLite_2.2.1            pls_2.7-3                broom_0.7.2
 [73] evaluate_0.14            stringr_1.4.0            yaml_2.2.1
 [76] bit64_4.0.5              zip_2.1.1                robustbase_0.93-6
 [79] ChemometricsWithR_0.2.0 purrr_0.3.4               nlme_3.1-149
 [82] formatR_1.7              R.oo_1.24.0              leaps_3.1
 [85] xml2_1.3.2               biomaRt_2.46.0           compiler_4.0.3
 [88] curl_4.3                 png_0.1-7                ggsignif_0.6.0
 [91] tibble_3.0.4             stringi_1.5.3            GenomicFeatures_1.42.1
 [94] forcats_0.5.0            Matrix_1.2-18            markdown_1.1
 [97] vctrs_0.3.5              pillar_1.4.7             lifecycle_0.2.0
[100] data.table_1.13.2        bitops_1.0-6             rtracklayer_1.49.5
[103] R6_2.5.0                 latticeExtra_0.6-29      hwriter_1.3.2
[106] rio_0.5.16               codetools_0.2-16         MASS_7.3-53
[109] assertthat_0.2.1         openssl_1.4.3            withr_2.3.0
[112] GenomeInfoDbData_1.2.4   diptest_0.75-7           mgcv_1.8-33
[115] hms_0.5.3                grid_4.0.3               tidyr_1.1.2
[118] class_7.3-17             rmarkdown_2.5            carData_3.0-4
[121] ggpubr_0.4.0             scatterplot3d_0.3-41
```
